# Neonatal Ventilation Training Using a Low-Cost, High-Fidelity Simulator in a Low-Resource Setting: A Randomized Clinical Trial

**DOI:** 10.1001/jamanetworkopen.2026.33261

**Published:** 2026-09-14

**Authors:** Davide Mocellin, Sabina Maglio, Mary-Winnie Nanyaro, Paolo Belardi, Martina Borellini, Elizabeth H. Shayo, Amerigo Ferrari, Benjamin Mfaume, Nyanda Ntinginya, Selene Tognarelli, Arianna Menciassi

**Affiliations:** 1 The BioRobotics Institute, Scuola Superiore Sant’Anna, Pisa, Italy; 2 Division of Obstetrics and Gynecology, Department of Clinical and Experimental Medicine, University of Pisa, Pisa, Italy; 3 Doctors with Africa CUAMM, Iringa, Tanzania; 4 Tosamaganga Regional Referral Hospital, Iringa, Tanzania; 5 National Institute for Medical Research, Dar es Salaam, Tanzania

## Abstract

**Importance:**

Neonatal mortality rates in sub-Saharan Africa remain high (26 per 1000 live births). Limited access to advanced respiratory support and shortage of trained health personnel make manual ventilation a lifesaving, yet high-risk, essential competence. Simulation-based training may strengthen neonatal ventilation skills, but evidence on acceptability and educational effectiveness of low-cost, high-fidelity simulators in resource-limited settings is limited.

**Objective:**

To build local capacity in manual neonatal ventilation and to assess acceptability and educational effectiveness of different simulator configurations across participants with varying levels of clinical experience.

**Design, Setting, and Participants:**

This randomized clinical trial was conducted from June 17 to 20, 2025, at a regional-level referral hospital in Tanzania. Participants included undergraduate medical students with no prior neonatal ventilation experience and local health professionals.

**Interventions:**

Participants were randomly assigned to 1 of 3 simulator configurations (medium-fidelity passive setup with no feedback, high-fidelity passive setup with mechanical feedback, and high-fidelity active setup with mechanical and sensors feedback). All participants received a 4-day training program consisting of theoretical lectures, hands-on simulation training with 1 of the 3 simulator configurations, and a standardized final evaluation.

**Main Outcomes and Measures:**

The primary outcome was objective ventilatory performance measured by the simulator during the hands-on sessions, defined as ventilation frequency and peak inspiratory pressure (PIP). Secondary outcomes included tutor-based performance evaluation, theoretical knowledge assessment in a 10-item questionnaire, and practical ventilation performance evaluation in a 12-item checklist. Tertiary outcomes were user-reported acceptability, perceived usefulness, perceived ease of use, behavioral intention, and simulator fidelity, assessed using the Technology Acceptance Model (TAM).

**Results:**

Both students (42 participants; median [IQR] age, 21 [20-22] years; 25 female [59.5%]) and health professionals (37 participants; median [IQR] age, 26 [23-29] years; 30 female [81.1%]) demonstrated improvements in theoretical knowledge (posttraining vs pretraining mean [SE] score [maximum 10 points]: students, 8.88 [0.13] vs 8.29 [0.17]; *P* < .001; health professionals, 9.14 [0.13] vs 8.65 [0.17]; *P* = .03) and practical ventilation performance following training (mean [SD] evaluation score [maximum 12 points]: students, 10.4 [0.7]; health professionals, 10.2 [0.9]). Training effectiveness varied by simulator configuration and participant experience. Among students, a medium-fidelity configuration was associated with improved ventilation frequency and PIP, whereas among health professionals, an active high-fidelity configuration supported improved performance during hands-on training. TAM scores confirmed a high simulator acceptability with no differences between groups.

**Conclusions and Relevance:**

In this randomized clinical trial of an adaptable, low-cost, high-fidelity neonatal ventilation simulator, effective training across heterogeneous learner groups in a resource-limited setting was supported; tailoring simulator feedback to learners’ baseline experience enhanced training effectiveness. These findings support the feasibility of scalable, simulation-based neonatal ventilation training in low-resource environments. Further research on skill retention, transfer to clinical practice, and neonatal outcomes are needed.

**Trial Registration:**

ClinicalTrials.gov Identifier: NCT07665164

## Key Points

**Question:**

Is training with a low-cost, high-fidelity neonatal ventilation simulator effective and acceptable for learners with different levels of clinical experience in a low-resource setting?

**Findings:**

In this randomized clinical trial including 42 medical students and 37 health professionals in Tanzania, simulation-based training was associated with improved knowledge and ventilation performance. Training effectiveness varied by simulator configuration and user experience, with simulator acceptability high across all groups.

**Meaning:**

These findings suggest that adaptable, low-cost, high-fidelity simulation platforms may support effective neonatal ventilation training and retraining for learners with different levels of experience in resource-limited settings.

## Teaser

This randomized clinical trial including medical students and health professionals in Tanzania examines the effectiveness of simulation-based training for neonatal ventilation techniques.

## Introduction

In 2023, data showed that neonatal mortality rate in sub-Saharan Africa (26 deaths per 1000 live births) was 8.7 times higher than in Europe and North America.^1^ The main causes of neonatal mortality are, in large part, attributable to preventable and treatable conditions occurring during birth.^1,2^ Evidence shows that the presence of skilled health personnel at delivery together with prompt access to emergency obstetric services and essential newborn care could prevent up to 40% of neonatal deaths.^3^ However, skilled birth attendance in sub-Saharan Africa remains drastically low, with only 40% of deliveries being assisted, compared with the 90% of the high-income countries.^1^ Strengthening the capacity of the health workforce to deliver essential, life-saving newborn care is therefore fundamental to ensure the delivery of quality-assured newborn care and reducing preventable neonatal deaths.^4,5,6^

Neonatal resuscitation is a time-sensitive, complex, and technically demanding intervention, requiring prompt decision-making under high-pressure conditions. Effective resuscitation primarily relies on timely initiation of positive-pressure ventilation and, when indicated, cardiopulmonary resuscitation.^7^ Across the African continent, substantial disparities persist in access to advanced respiratory support technologies for neonatal ventilation, as well as in the availability of adequately trained health care personnel.^8^ As a result, manual ventilation with self-inflating bags constitutes the primary and often the only method of respiratory support.

Manual ventilation is a highly operator-dependent procedure that can cause substantial health risks for the neonate when inappropriately performed.^9,10^ In resource-scarce settings, where access to advanced respiratory support technologies remains limited, technical proficiency in manual ventilation is essential for safe and effective neonatal care.^11,12,13^

Standardized neonatal resuscitation training programs have been implemented in sub-Saharan Africa, and were associated with improvements in clinician performance and competence, team coordination, and error mitigation.^14,15,16^ Accordingly, current international guidelines recommend the use of simulation-based training in neonatal resuscitation and support its implementation in low- and middle-income countries.^17,18,19^

In sub-Saharan Africa, low-cost, high-fidelity simulators have gained attention for their ability to provide a realistic replication of clinical procedures while maintaining affordability and accessibility.^7,20,21^ Additionally, evidence suggests that the combination of such simulators with locally available resources and trained facilitators represents an effective and sustainable approach for developing neonatal resuscitation training programs tailored to low-resource settings.^22,23^ Despite this growing evidence, data on the implementation, acceptability, and teaching effectiveness of such training interventions in clinical settings remain limited.

In this study, we report the implementation and assessment of a simulation-based neonatal ventilation training intervention delivered to a heterogeneous cohort of Tanzanian medical students and health care professionals at a regional-level hospital in Tanzania. Our aim was to build local capacity in manual neonatal ventilation, through both lectures and hands-on training using a new low-cost, high-fidelity simulator, and to assess the acceptability and educational effectiveness of different simulator configurations across participants with varying levels of clinical experience. Specifically, the objectives of the study were (1) to evaluate the educational effectiveness of different simulator configurations across participants with varying levels of clinical experience, using objective performance metrics collected by the simulator; (2) to assess the acquisition of core manual ventilation competencies among inexperienced medical students through objective tutor evaluation and knowledge assessment; (3) to assess skill reinforcement among experienced clinicians through objective tutor evaluation and knowledge assessment, and; (4) to examine user-reported usability and perceived usefulness of the simulator configurations.

## Methods

### Study Setting

This randomized clinical trial was conducted at Tosamaganga Regional Referral Hospital (TRRH), a nonprofit, faith-based facility owned by the regional Catholic diocese that operates as a regional-level referral center managing a high volume of deliveries and major obstetric emergencies in the Iringa region of Tanzania (trial protocol and statistical analysis plan available in Supplement 1).^24^ The hospital includes the Tosamaganga Institute of Health and Allied Sciences (TIHAS) for health workers training. Doctors with Africa CUAMM (CUAMM) is an international nongovernmental organization that has been supporting TRRH for decades in various medical areas, including maternal and newborn health. This study was conducted through a collaboration among TRRH, CUAMM, the TIHAS, the Sant’Anna School of Advanced Studies (Italy) (SSSA), and the National Institute for Medical Research (NIMR).

### Neonatal Ventilation Simulator and Equipment

The neonatal ventilation simulator was developed by SSSA with the aim to replicate neonatal airway anatomy and ventilation mechanics and to provide objective performance feedback. The system integrates a physical manikin with pressure sensors and a mobile user interface enabling real-time data acquisition and providing visual and auditory feedback to the trainee (eFigure 1 in Supplement 2). A detailed technical description is provided in eMethods in Supplement 2. The equipment used during the hands-on training consisted of: 2 small cotton blankets, a newborn penguin suction device, a newborn self-inflating bag with a size 1 anatomical mask, gloves, and a small wool hat.

### Study Population and Group Allocation

Two study cohorts were enrolled: second-year undergraduate students attending the clinical officer training program at TIHAS, and nurses, midwives, clinical officers, and pediatrics working at Tosamaganga Hospital. The students did not have prior experience in neonatal ventilation. This choice was made to have the maximum possible impact with the training. Written informed consent was obtained from all participants prior to enrolment.

**Figure 1.**
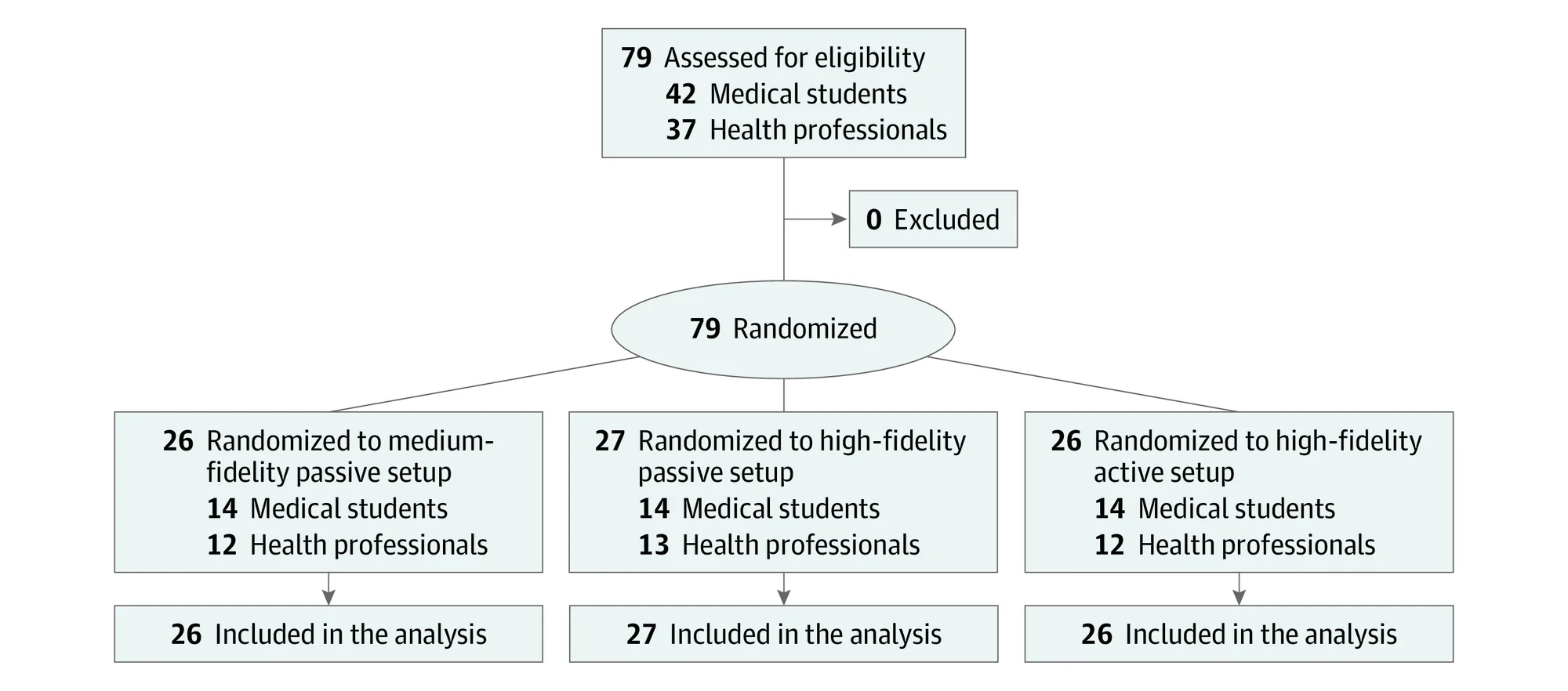
Participant Recruitment Flowchart

Participants were randomly assigned to 1 of the 3 groups corresponding to different simulator configurations, with balanced group sizes (3 students groups: 14, 14, and 14; 3 health professionals groups: 12, 13, and 12, respectively) (Figure 1). The first group trained with a medium-fidelity passive setup, which did not have mechanical feedback or mobile user interface guidance. The second group trained with a high-fidelity passive setup providing mechanical feedback through chest and/or abdominal rise, without mobile user interface guidance. The third group trained with a high-fidelity active setup combining mechanical feedback with real-time, sensor-based guidance via a mobile user interface (eFigure 1 in Supplement 2).

Passive configurations did not include electronic or software-based feedback, whereas the active configuration provided immediate guidance through electronic components and both visual and auditory feedback. Simulator-derived data were collected and stored in all configurations for subsequent performance analysis.

Based on an a priori power calculation (power, 80%; α = .05), a minimum of 8 participants per group were required. This number was increased to 10 to account for a 20% potential nonresponse rate (eMethods in Supplement 2).

### Ethics, Data Protection, and Reporting

Ethical approval was obtained from the National Health Research Ethics Committee of NIMR (approval No. number NIMR/HQ/R.8a/Vol.IX/4983). All data were anonymized and handled in accordance with institutional and national data protection regulations. Voluntary participation was key, and all study participants signed a written informed consent form. Additional information is provided in eMethods in Supplement 2. The randomized clinical trial was registered on ClinicalTrial.gov (NCT07665164) and follows the Consolidated Standards of Reporting Trials (CONSORT) reporting guideline.^25^

### Training Procedure

The training course was delivered over 4 consecutive days (June 17 through June 20, 2025) and combined lectures, hands-on simulation training, and final performance evaluation. To accommodate the number of participants within the planned timeframe, 2 identical simulators were used in parallel. On the first day of training, participants took part in preassessment surveys and lectures. All participants completed a pretraining survey to assess baseline knowledge and a sociodemographic survey (eTables 14 and 15 in Supplement 2). Participants also attended half-day standardized lectures in which the teacher illustrated fundamental theoretical concepts regarding the neonatal manual emergency ventilation and the principles of simulation in medical training.

The second and third days were dedicated to demonstration and hands-on training. Each day, was dedicated to one hands-on training session (according to randomization). Hands-on sessions began with a brief clinical procedure demonstration, then each participant had a 1-minute familiarization period with the simulator followed by three 1-minute ventilation trials. In each trial, participants were required to position the newborn, place the mask, and deliver the manual ventilation at a frequency of 40 breaths/min and peak inspiratory pressure of 25 cm H_2_O.^19^ There was a minimum 24-hour washout period between sessions. Overall, each participant completed a total of 6 hands-on ventilation trials divided into 2 sessions of 3 trials.

On day 4, assessment and posttraining surveys were administered. All participants completed a standardized evaluation session performing a 1-minute ventilation using the simulator configured in a high-fidelity passive mode, mimicking the real clinical conditions where the chest rise is the only available feedback. Similarly to the training session, participants were required to position the newborn, place the mask, and deliver the manual ventilation at target frequency and pressure.^19^ A tutor collected data through the mobile user interface. Performance was objectively assessed by an external tutor using a quantitative evaluation sheet (eTable 16 in Supplement 2) and simulator-derived data. Following the evaluation session, participants completed 2 posttraining surveys: one assessing the acquired knowledge (eTable 14 in Supplement 2), and one assessing the acceptability of the simulator (eTable 17 in Supplement 2). The assessment and posttraining surveys required a maximum of 15 minutes per participant. The external tutor was unfamiliar to the participants and completed the quantitative evaluation sheets during the assessments to minimize potential recall and observer bias.

### Outcome Measures

The primary outcome (used to assess objective 1), was the ventilatory performance measured by the simulator, ie, ventilation rate (breaths per minute [BPM]) and peak inspiratory pressure (PIP). In accordance with international guidelines, we considered as criterion standard a ventilation rate of 40 breaths/min and a PIP of 25 cm H_2_O.^19^ For each participant and trial, we computed the mean and standard deviation of both indicators (BPM and PIP). The secondary outcomes (objectives 2 and 3), were acquisition and reinforcement of manual ventilation knowledge and skills, assessed through: (1) data from the simulator sensors acquired during the final evaluation session, (2) objective tutor-based performance evaluation using a structured quantitative scoring sheet on a 3-point scale (with 0 graded as poor, 0.5 as average, and 1 as good) (eTable 16 in Supplement 2), (3) theoretical knowledge assessment (pretraining and posttraining true or false questionnaire with a maximum 10 point score; along with a scored 12-step checklist on practical ventilation performance) (eTable 14 in Supplement 2).

The tertiary outcomes (objective 4) were user-reported measures of simulator acceptability over the 4 dimensions of the Technology Acceptance Model (TAM) scale: perceived usefulness, perceived ease of use, behavioral intention, and fidelity of the simulator.^26,27^ The items were tailored to the characteristics of the simulator and rated on 7-point Likert scales (from “extremely disagree” to “extremely agree”) (eTable 15 in Supplement 2).

### Statistical Analysis

Simulator-derived sensor data, tutor assessments, and survey responses were recorded and transcribed into electronic spreadsheets containing only unique numeric participant identifiers. The unique identifiers were used for data linkage between the different datasets collected.

The sociodemographic characteristics of the participants, expressed as numbers and percentages (categorical variables) or as medians and interquartile ranges (continuous variables), were compared between the 3 simulation groups using the χ^2^ test for categorical variables or the Kruskal-Wallis test for continuous variables to verify homogeneity between groups.

To evaluate the longitudinal evolution of ventilation performance across the 6 training attempts (trials 1 through 6), we fitted linear mixed-effects models separately for each outcome: mean peak inspiratory pressure (PIP), ventilation rate (BPM), and their corresponding coefficients of variation (CV; hereafter PIP CV and BPM CV) computed as the ratio of the standard deviation to the mean. The main estimand for this model was the within-group learning trajectory across trials, using trial 1 as the reference point to characterize individual group progression. Trial (1 through 6) and group assignment (medium-fidelity passive, high-fidelity passive, high-fidelity active) were included as categorical fixed effects, together with their interaction term, to examine whether performance trajectories differed between groups.

Models were estimated by using maximum likelihood under the assumption of normally distributed random effects. After model fitting, adjusted marginal means (projected means) and 95% CIs were computed. These margins were plotted across trials for each group to visualize the temporal evolution of performance.

For the secondary outcomes simulator data and tutor evaluation, we used a comparison between the 3 groups using the ANOVA test (after verifying the normal distribution of continuous variables through the Shapiro-Wilk test) and for the theoretical knowledge assessment we used the Wilcoxon test for paired samples. The main estimand here was the between-group difference in final competency at the assessment, which serves as a distinct benchmark from the longitudinal training data.

For the tertiary outcome, we ran a partial least squares structural equation model (PLS-SEM) to test both the associations between the TAM items and the respective dimensions considered as latent variables (measurement model) and 5 hypothesized relationships (structural model): (1) fidelity of the simulator improves perceived usefulness; (2) perceived ease of use improves perceived usefulness; (3) perceived usefulness improves behavioral intention; (4) fidelity of the simulator improves perceived ease of use, and; (5) fidelity of the simulator improves behavioral intention.

Data management and statistical analyses were performed using Stata Software version 17.0 (StataCorp LLC). Hypothesis tests were 2-sided, with significance set at *P* < .05. Further statistical considerations can be found in the eMethods in Supplement 2.

## Results

### Baseline Characteristics of the Study Populations

A total of 79 participants were enrolled, including 37 health professionals and 42 medical students. Among students, the median (IQR) age was 21 (20-22) years; 25 were female (59.5%) and 17 male (40.5%); all were training as clinical officers. Among health professionals, the median (IQR) age was 26 (23-29) years; 30 were female (81.1%) and 7 male (18.9%). Most participants were midwives (29 [78.4%]). The majority of participants in both groups reported being previously involved in some hands-on training (28 students [66.7%], 34 health professionals [91.9%]) and to have a clinical background (33 students [78.6%], 35 health professionals [94.6%]). No significant differences in baseline characteristics were observed among the 3 study groups within either population. Detailed baseline characteristics of students and health professionals are reported in eTables 1 and 2 in Supplement 2.

### Objective 1: Educational Effectiveness of Different Simulator Configurations

#### Results by Students Groups

**Figure 2.**
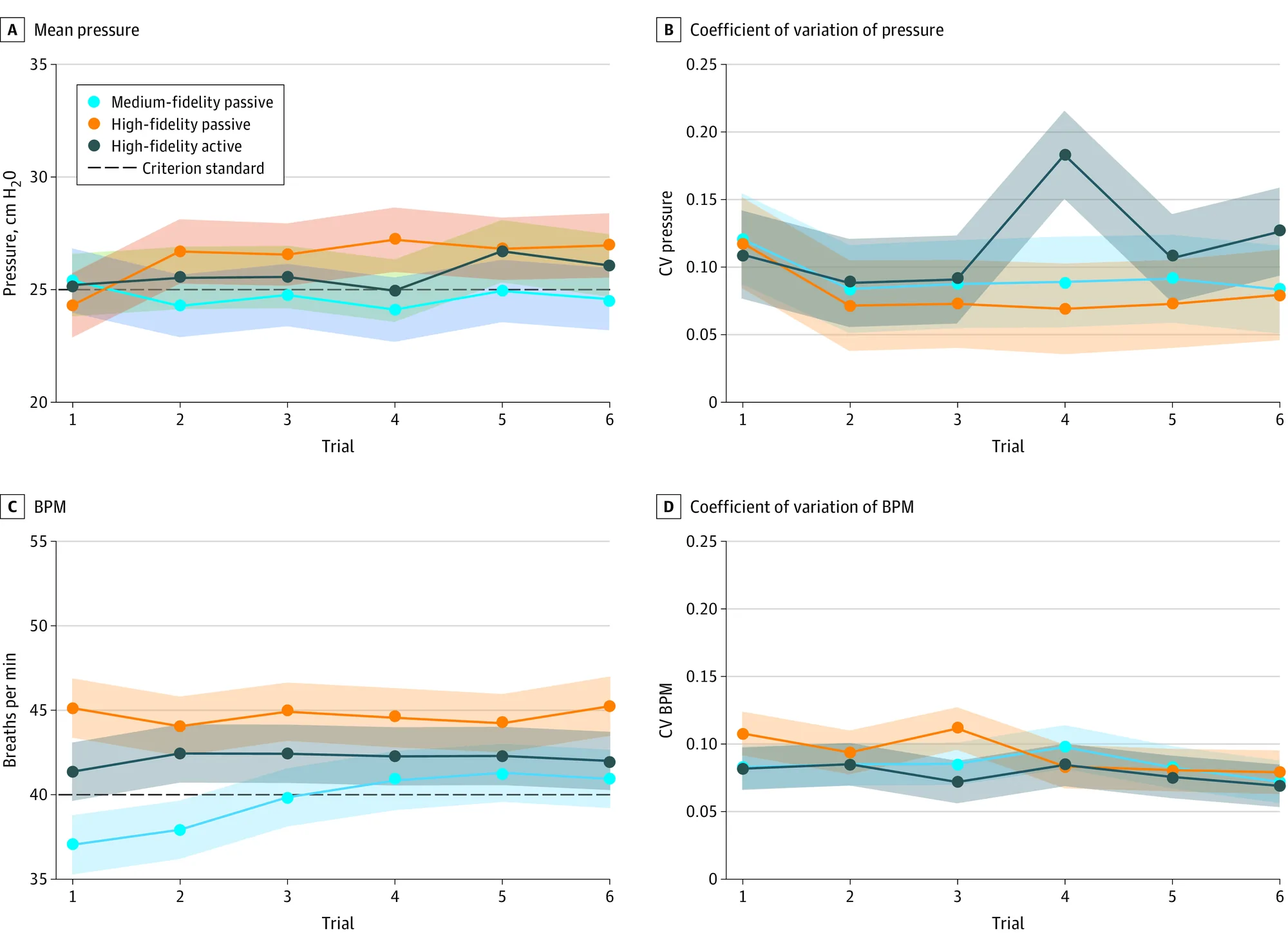
Line Graph of Simulator Results Across the 6 Trials of Hands-on Sessions for Student Population Shaded areas indicate 95% CIs; BPM, breaths per minute; CV, coefficient of variation. The criterion standard for pressure is 25 cm H_2_O, for BPM is 40 breaths/min.

The linear mixed-effects models revealed distinct overall performance trajectories and group-by-trial interaction patterns across the 6 training sessions. Across the 6 trials, the overall interaction model for mean airway pressure showed that the high-fidelity passive group showed a significant decline in performance, failing to reach the target pressure in all trials after trial 1; trial-specific comparisons relative to baseline tracked this progression (mean pressure: trial 1, 24.3 cm H_2_O [95% CI, 22.9-25.7 cm H_2_O]; trial 2, 26.7 cm H_2_O [95% CI, 25.2-28.1 cm H_2_O]; trial 3, 26.5 cm H_2_O [95% CI, 25.2-28.0 cm H_2_O]; trial 4, 27.2 cm H_2_O [95% CI, 25.8-28.6 cm H_2_O]; trial 5, 26.8 cm H_2_O [95% CI, 25.4-28.2 cm H_2_O]; trial 6, 27.0 cm H_2_O [95% CI, 25.5-28.4 cm H_2_O]) (Figure 2A). The medium-fidelity passive and high-fidelity active groups reached the target in most trials and showed no significant changes in performance relative to trial 1; the only exception was trial 5 in the high-fidelity active group, which did not differ significantly from baseline (mean pressure, 26.7 cm H_2_O [95% CI, 25.3-28.1 cm H_2_O]; *P* = .08).

Analysis of pressure variability indicated a significant longitudinal reduction in the coefficient of variation (PIP CV) for the high-fidelity passive group, with secondary trial-by-trial analysis demonstrating this shift in trials 2 through 5 compared with trial 1 (PIP CV per trial: trial 1, 0.12 [95% CI, 0.09-0.15]; trials 2 through 5, 0.7 [95% CI, 0.04-0.11]) (Figure 2B; eTable 4 in Supplement 2). In contrast, the high-fidelity active group showed increased pressure variability at trial 4 (PIP CV per trial: trial 1, 0.11, trial 4, 0.18), while no significant changes were observed for the medium-fidelity passive group.

Regarding breathing frequency, the mixed-effects model identified a significant overall improvement trend only in the medium-fidelity passive group, reaching the target ventilation rate in trials 3 through 6 compared with trials 1 and 2 (mean BPM per trial: trial 1, 37.0 breaths/min [95% CI, 35.2-38.7 breaths/min]; trial 2, 38.0 breaths/min [95% CI, 36.1-39.5 breaths/min]; trial 3, 40.0 breaths/min [95% CI, 38.1-41.5 breaths/min]; trial 4, 40.8 [95% CI, 39.0-42.5 breaths/min]; trial 5, 41.2 [95% CI, 39.5-43.0 breaths/min]; trial 6, 40.9 breaths/min [95% CI, 39.1-42.6 breaths/min]) (Figure 2C; eTable 5 in Supplement 2). The high-fidelity passive group did not show significant changes and failed to reach the target in all trials. The high-fidelity active group reached the target only at trial 1, with no significant changes thereafter (mean BPM per trial in breaths per minute: trial 1, 41.3 breaths/min [95% CI, 39.6-43.0 breaths/min]).

Variability in breathing frequency decreased significantly as an overall pattern only in the high-fidelity passive group in trials 4 through 6 compared with trial 1 (BPM CV per trial: trial 1, 0.11 [95% CI, 0.09-0.12]; trials 4 through 6, 0.08 [95% CI, 0.07-0.10]); no significant changes were observed in the medium-fidelity passive and high-fidelity active groups across the 6 trials (Figure 2D; eTable 6 in Supplement 2).

#### Results by Health Professionals Groups

**Figure 3.**
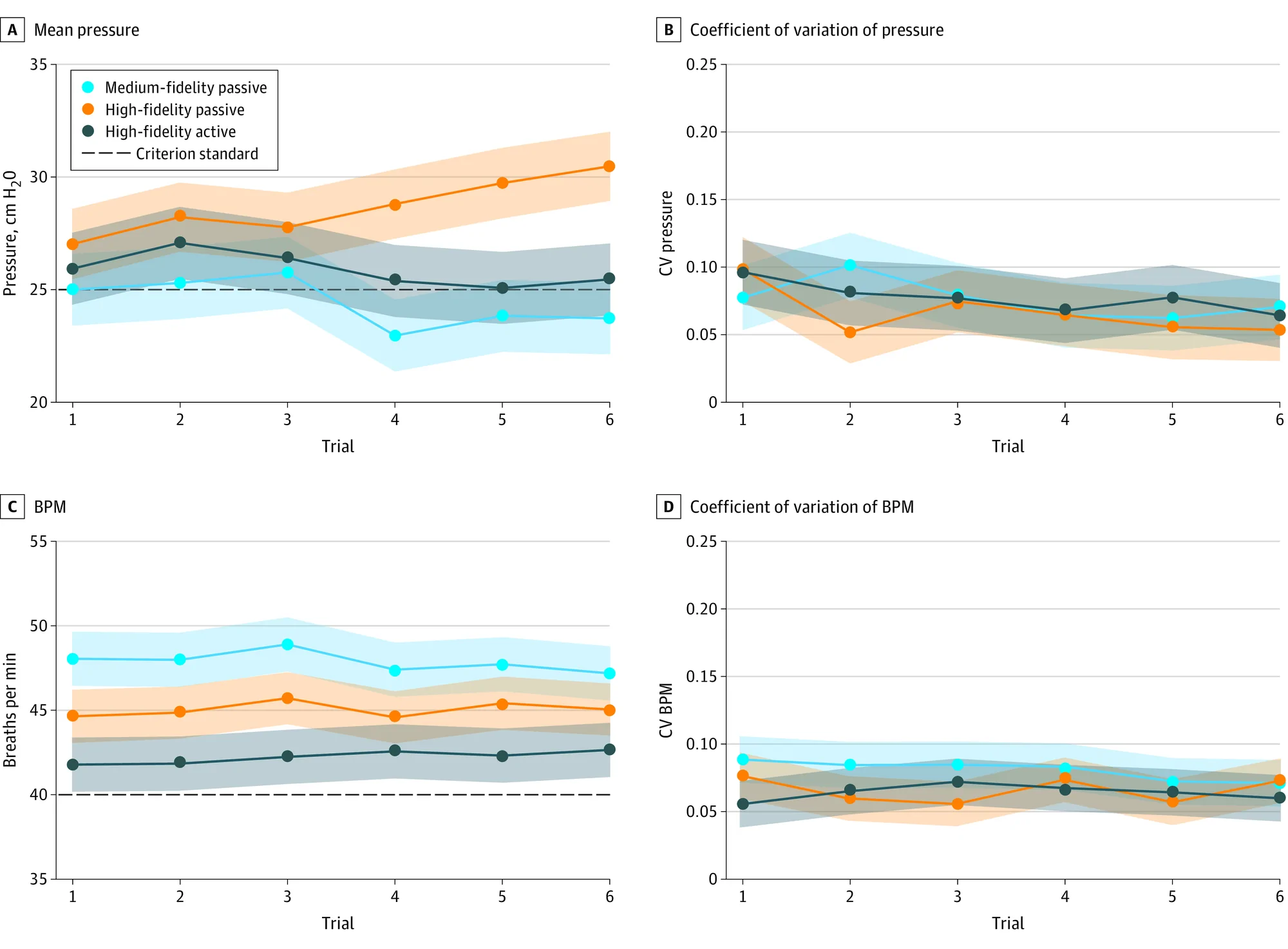
Line Graph of Simulator Results Across the 6 Trials of Hands-on Sessions for Health Professionals Population BPM indicates breaths per minute; CV, coefficient of variation. Shaded area indicates 95% CIs. The criterion standard for pressure is 25 cm H_2_O, for BPM is 40 breaths/min.

For experienced health professionals, the mixed-effects models showed more stable baseline behaviors but highly specific group-by-trial interaction patterns. As measured by mean pressure, the medium-fidelity passive group demonstrated a significant decrease in performance at trial 4 (mean pressure per trial: trial 1, 25.0 cm H_2_O [95% CI, 23.3-26.6 cm H_2_O]; trial 4, 23.0 cm H_2_O [95% CI, 21.3-24.5 cm H_2_O]), failing to reach the target pressure of 25.0 cm H_2_O, while meeting the target in all other trials (Figure 3A; eTable 7 in Supplement 2). However, the global pattern for the high-fidelity passive group, showed a progressive upward trend, with significantly higher pressures in trials 4 through 6 compared with trial 1 (mean pressure per trial: trial 1, 27.0 cm H_2_O [95% CI, 25.4-28.6 cm H_2_O]; trial 4, 28.8 cm H_2_O [95% CI, 27.2-30.3 cm H_2_O]; trial 5, 29.7 cm H_2_O [95% CI, 28.1-31.3 cm H_2_O]; trial 6, 30.4 cm H_2_O [95% CI, 28.9-32.0 cm H_2_O]) and did not reach the target in any trial. The high-fidelity active group failed to reach the target only in trial 2 (mean pressure in trial 2, 27.0 cm H_2_O [95% CI, 25.4-28.6 cm H_2_O]) and exhibited no significant changes across trials.

Analysis of pressure variability indicated a significant overall reduction in the CV of pressure for the high-fidelity passive group, with trial-specific data indicating improvements in trials 2, 4, 5, and 6 (PIP CV per trial: trial 1, 0.10 [95% CI, 0.07-0.12]; trial 2, 0.05 [95% CI, 0.03-0.07]; trial 4, 0.06 [95% CI, 0.04-0.09]; trial 5, 0.06 [95% CI, 0.03-0.08]; trial 6, 0.05 [95% CI, 0.03-0.08]) (Figure 3B; eTable 8 in Supplement 2). The high-fidelity active group showed improved pressure consistency in trials 4 and 6 (PIP CV per trial: trial 1, 0.10 [95% CI, 0.08-0.13]; trial 4, 0.07 [95% CI, 0.04-0.09]; trial 6, 0.06 [95% CI, 0.04-0.09]), whereas no significant changes were observed for the medium-fidelity passive group.

The mixed model confirmed a lack of overall systemic changes for breathing frequency; no group achieved the target ventilation rate of 40 breaths/min, and mean BPM did not differ significantly across trials (Figure 3C; eTable 9 in Supplement 2). The high-fidelity active group was closest to the target (41.7 breaths/min [95% CI, 40.2-43.4 breaths/min] in trial 1), whereas the medium-fidelity passive group showed the highest mean rate (48.8 breaths/min [95% CI, 47.3-50.5 breaths/min] in trial 3). Variability in breathing frequency decreased significantly only in the high-fidelity passive group at trial 3 compared with trial 1 (BPM CV per trial: trial 1 = 0.08; trial 3 = 0.06; *P* = .04), with no significant changes observed in the medium-fidelity passive and high-fidelity active groups (Figure 3D).

### Objective 2: Acquisition of Core Manual Ventilation Competencies Among Students

The acquisition of core manual ventilation competences was assessed with 3 instruments: the pre- and posttraining theoretical test, the objective evaluation of the performance during the final test made by the tutor, and the PIP and BPM measured by the simulator during the final evaluation session.

Among medical students, a significant improvement was observed in theoretical knowledge following training, with higher posttraining scores compared with pretraining scores (mean [SE], 8.88 [0.13] vs 8.29 [0.17] of a maximum 10 score; *P* < .001). At the final assessment, objective tutor evaluation scores did not differ significantly across simulator configurations (mean [SD] evaluation [maximum 12 score], 10.4 [0.7]) (eTable 11 in Supplement 2). Similarly, no significant differences between groups were observed in mean PIP or BPM. The medium-fidelity passive group showed a mean BPM closer to the reference value of 40 breaths/min compared with the high-fidelity groups, although this difference was not statistically significant.

### Objective 3: Skill Reinforcement Among Experienced Health Professionals

Among experienced health professionals, posttraining theoretical test scores were significantly higher than pretraining scores (mean [SD] on 10-point scale, 9.14 [0.13] vs 8.65 [0.17]; *P* = .03). Final tutor evaluation scores did not differ significantly among simulator configurations (mean [SD] evaluation on 12-point scale, 10.2 [0.9]) (eTable 12 in Supplement 2). Mean PIP values during the final test were higher than the reference value (25 cm H_2_O) in all groups, with no significant between-group differences (mean [SD] PIP: medium-fidelity passive, 27.1 (4.1) cm H_2_O, high-fidelity passive, 30.1 (7.9) cm H_2_O; high-fidelity active, 28.5 (3.4) cm H_2_O; *P* = .11). In contrast, a significant difference was observed in mean BPM across groups (mean [SD] BPM: medium-fidelity passive group, 42.8 [7.0] breaths/min; high-fidelity passive group, 39.9 [8.3] breaths/min; high-fidelity active group, 48.8 [8.9] breaths/min; ANOVA *P* = .04), with the high-fidelity passive group achieving values closest to the reference rate of 40 breaths/min. Post hoc Bonferroni analysis identified a significant difference between high-fidelity passive and high-fidelity active groups (*P* = .04), whereas no differences were observed between the medium-fidelity passive and high-fidelity passive groups or the medium-fidelity passive and high-fidelity active groups (eTable 13 in Supplement 2).

### Objective 4: User-Reported Usability and Perceived Usefulness of the Simulator Configurations

The measurement model demonstrated adequate reliability and validity in both health professionals and student populations, with factor loadings above recommended thresholds (0.7) for all items except perceived usefulness, and satisfactory composite reliability, Cronbach α, ρ A, and average variance extracted values (Tables 1 and 2).

**Table 1.** PLS-SEM of the TAM Survey for the Population of Students^a^

Latent variable	Indicator variable	Loading	Cronbach α	Composite reliability	ρ A
Measurement model					
PU	PU1	0.781	0.815	0.869	0.839
	PU2	0.598			
	PU3	0.820			
	PU4	0.770			
	PU5	0.795			
PEU	PEU1	0.759	0.844	0.887	0.850
	PEU2	0.782			
	PEU3	0.769			
	PEU4	0.823			
	PEU5	0.776			
BI	BI1	0.827	0.778	0.793	0.780
	BI2	0.794			
FS	FS1	0.956	0.900	0.938	0.905
	FS2	0.887			
	FS3	0.895			
Reference values for validity	NA	>0.7	>0.7	>0.7	Internally consistent^b^

Abbreviations: BI, behavioral intention; FS, fidelity of the simulator; NA, not applicable; PEU, perceived ease-of-use; PLS-SEM, partial least squares structural equation model; PU, perceived usefulness; TAM, Technology Acceptance Model.

^a^ The loadings of all TAM items (PU1, PU3, PU4, PU5, PEU1, PEU2, PEU3, PEU4, PEU5, BI1, BI2, FS1, FS2, except PU2) were >0.7, confirming that all items serve as reliable indicators of the measured latent variables.

^b^ The model fitting was confirmed by all values of composite reliability and Cronbach α >0.7, and by ρ A values between composite reliability and Cronbach α. The reliability coefficient ρ A provides an estimate that typically falls between Cronbach α (which tends to underestimate reliability) and composite reliability (which tends to overestimate it, since it assumes equal indicator loadings). A ρ A value within this range, and generally above 0.70, indicates that the construct’s reliability estimate is neither too conservative nor too liberal, supporting adequate internal consistency of the measurement model. In our analysis, ρ A was calculated for each latent construct individually as part of the internal consistency reliability assessment.

**Table 2.** PLS-SEM of the TAM Survey for the Population of Health Professionals^a^

Latent variable	Indicator variable	Loading	Cronbach α	Composite reliability	ρ A
PU	PU1	0.761	0.829	0.876	0.854
	PU2	0.650			
	PU3	0.838			
	PU4	0.822			
	PU5	0.747			
PEU	PEU1	0.737	0.890	0.920	0.904
	PEU2	0.728			
	PEU3	0.895			
	PEU4	0.893			
	PEU5	0.904			
BI	BI1	0.901	0.834	0.922	0.889
	BI2	0.947			
FS	FS1	0.815	0.830	0.897	0.856
	FS2	0.881			
	FS3	0.888			
Reference values for validity	NA	>0.7	>0.7	>0.7	Internally consistent^b^

Abbreviations: BI, behavioral intention; FS, fidelity of the simulator; NA, not applicable; PEU, perceived ease-of-use; PLS-SEM, partial least squares structural equation model; PU, perceived usefulness; TAM, Technology Acceptance Model.

^a^ The loadings of all TAM items (PU1, PU3, PU4, PU5, PEU1, PEU2, PEU3, PEU4, PEU5, BI1, BI2, FS1, FS2, except PU2) were >0.7 confirming that all items serve as reliable indicators of the measured latent variables.

^b^ The model fitting was confirmed by all values of composite reliability and Cronbach α >0.7, and by ρ A values between composite reliability and Cronbach α. The reliability coefficient ρ A provides an estimate that typically falls between Cronbach α (which tends to underestimate reliability) and composite reliability (which tends to overestimate it, since it assumes equal indicator loadings). A ρ A value within this range, and generally above 0.70, indicates that the construct’s reliability estimate is neither too conservative nor too liberal, supporting adequate internal consistency of the measurement model. In our analysis, ρ A was calculated for each latent construct individually as part of the internal consistency reliability assessment.

Structural model analysis identified perceived usefulness as a significant factor in estimating behavioral intention in both health professionals and students, with perceived ease of use positively associated with perceived usefulness (eTable 18 in Supplement 2). Fidelity of simulation was significantly associated with perceived ease of use in both populations. However, fidelity was associated with behavioral intention only among health professionals, whereas this was not observed in students. Fidelity was not significantly associated with perceived usefulness in either group. Comparison of latent variables across simulator configurations using the Kruskal-Wallis test revealed no statistically significant differences between groups in either population.

## Discussion

Our study describes the implementation and validation of a simulation-based neonatal ventilation training delivered to medical students and health care professionals at the TRRH in Tanzania. The study investigated the use of a low-cost high-fidelity simulator with 3 different modalities of real-time feedback.

### Acquisition of Core Manual Ventilation Competencies

The final evaluation demonstrated high levels of theoretical and practical proficiency, with all participants achieving positive final scores in the theoretical test (higher than 8 of 10) and in the objective evaluation conducted by the tutor (above 9.8 of 12). While the high baseline scores of the theoretical test resulted in a ceiling effect that limited the magnitude of measurable improvement, these findings demonstrate the educational effectiveness of the simulation and the teaching methods employed and are consistent with prior evidence supporting simulation-based training as an effective method for consolidating theoretical knowledge, educating novice medical students and reinforcing skills in experienced clinical staff.^22,23,28,29^

### Educational Effectiveness of Different Simulator Configurations

Among students, the best performance during the practical training phase was observed in the medium-fidelity passive group. This group consistently achieved the target pressure across all trials and showed a significant improvement in ventilation rate, reaching correct ventilation technique by the end of the first training day and maintaining it on the second day. The high-fidelity active group was able to follow the simulator’s visual feedback to achieve the target pressure but had difficulty following the auditory feedback to maintain an appropriate ventilation rate. The high-fidelity passive group, which received only mechanical feedback from chest rise, exhibited overventilation, applying both pressures and ventilation rates above the target.

At the final assessment, no significant differences were observed among the 3 student groups, although the medium-fidelity passive group again demonstrated ventilation values closer to the target. These findings suggest that students without prior experience may learn more effectively when exposed to limited stimuli, as in medium-fidelity simulation, allowing greater focus on instructor guidance and task execution, thereby facilitating motor learning and skill acquisition.

Among health professionals, the best performance during practical training was observed in the high-fidelity active group, which achieved the target pressure in most trials, improved pressure consistency, and more closely approached the target ventilation rate compared with the other groups. Performance remained stable across trials, with further improvement in mean delivered pressure on the second training day, suggesting adaptation to combined visual and auditory feedback. In contrast, the medium-fidelity and high-fidelity passive groups showed greater variability and poorer performance in both pressure and breathing frequency. The high-fidelity passive group exhibited persistent overventilation, applying pressures and ventilation rates above target values, with elevated pressures maintained during the final test.

At the final assessment, no significant differences in mean pressure were observed among health professionals groups; however, the high-fidelity active group showed a deterioration in ventilation frequency, deviating from the target, which resulted in a significant post hoc difference compared with the high-fidelity passive group, whose participants maintained values closer to the reference rate. This discrepancy between training and final assessment may reflect examination-related anxiety, differences in testing conditions, and the absence of auditory feedback during the examination where the high-fidelity passive set-up of the simulator, with only mechanical feedback, was used. Overall, although the high-fidelity active group benefited most from simulator feedback during hands-on training, these gains were not fully transferred to the final assessment, whereas the other groups did not achieve optimal ventilation performance during either training or testing, suggesting that simulator feedback may be particularly relevant for expert health professionals retraining.

These findings are consistent with prior research indicating that high-fidelity simulation provides greater benefit for the hands-on training of clinically experienced medical personnel, whereas medium-fidelity simulation is more effective for training unskilled medical students.^30,31,32,33^ Furthermore, international guidelines^19^ suggest that during manual ventilation, practitioners should initiate PIP at 20 to 25 cm H_2_O, which can be raised up to a maximum of 40 cm H_2_O, with a ventilation frequency of 40 to 60 breaths/min.^19^ Both students and health care professionals maintained these ranges throughout all training trials and the final evaluation, with the exception of the first 2 training trials in the student medium-fidelity passive group, where the breathing frequency fell below 40 breaths/min. Based on these observations, it is evident that all groups achieved a clinically satisfactory performance during both the training phase and the final assessment.

### User-Reported Usability And Perceived Usefulness Of The Simulator Configurations

Technology acceptance analysis revealed both shared and population-specific factors associated with simulator adoption. In both students and health professionals, perceived usefulness was a significant predictor of behavioral intention, and perceived ease-of-use was positively associated with influenced perceived usefulness, indicating that usability is linked to acceptance across experience levels. Simulation fidelity was consistently associated with perceived ease of use, suggesting that realistic simulator behavior is accompanied by more intuitive interaction. However, fidelity did not directly influence perceived usefulness in either group. Notably, simulation fidelity was significantly associated with behavioral intention only among health professionals, indicating that fidelity plays a critical motivational role in experienced clinicians but not in novices. Together, these findings suggest that usability and perceived usefulness are strongly related to acceptance for all users, whereas high-fidelity features may be particularly important for engaging and retraining experienced clinical staff as shown in previous studies.^22,23,34,35^

The newly developed simulator from the Sant’Anna School supported training across a heterogeneous cohort, benefiting both medical students and experienced health professionals, reflecting real-word target population for neonatal ventilation training. Its configurable design allows modulation of real-time feedback, enabling adaptation of training to users’ baseline skill levels and supporting progressive and targeted skill development. Together with low cost, high fidelity, ease of use, and limited maintenance requirements, these features address key barriers to the implementation of simulation-based training in resource-scarce settings. Such adaptable simulation platforms may facilitate scalable neonatal ventilation training across diverse learner groups and support sustainable training and retraining pathways in sub-Saharan Africa. Beyond individual skill acquisition, these tools may contribute to standardization of training approaches and strengthening of health workforce capacity without reliance on complex infrastructure. Moreover, the study underscores the role of interinstitutional collaboration in developing context-appropriate simulation technologies for low-resource health care systems. Future studies should evaluate transfer to clinical practice, long-term skill retention, and integration into routine training programs.

### Limitations

This study has some limitations. The single-center design may have limitations in the generalizability of the findings. Not considered residual confounding variables cannot be excluded. Although no baseline differences were observed among groups, randomization may have resulted in imbalanced participants characteristics that we did not account for. The sample size was limited, which may have reduced the statistical power to detect smaller but potentially meaningful differences between simulator configurations. The final assessment was performed using the high-fidelity passive setup to best replicate clinical reality. While this standardized approach prioritized clinical validity, it inherently favored participants who trained with this configuration. We acknowledge this as a limitation in our comparative design, as the study focuses on skill transfer to a clinical neonatal context rather than performance within training-specific environments. This study was conducted in a controlled simulation-based environment, and the findings should therefore be interpreted within this context. The results are limited to simulated performance and do not allow conclusions regarding real-world clinical practice or patient outcomes. In addition, no data were collected to evaluate whether the training effects translate into improvements in clinical metrics or address the clinical challenges encountered in African health care settings. Finally, outcome measures based on simulator-derived metrics and tutor assessment may not capture all clinically relevant aspects of manual ventilation performance. Specifically, our evaluation relied on absolute performance deviation from precise numerical targets rather than clinical range-based metrics (such as the percentage of breaths within a target window), which may limit our immediate insights into how novice technical precision maps onto broader clinical safety margins.

## Conclusions

In this randomized clinical trial of simulation-based neonatal ventilation training, the use of a low-cost, high-fidelity simulator was associated with acquisition and reinforcement of manual ventilation skills among both medical students and experienced health professionals in a resource-limited setting. Training performance differed according to simulator configuration and participant experience, indicating that simulation modalities should be tailored to learners’ baseline skills. User-reported simulator acceptance supported the feasibility of implementing this approach in low-resource settings. Further studies are needed to investigate the transition from technical precision to clinical range-based safety margins, and to assess skill retention, transfer to clinical practice, and patient outcomes.

## Notes

Supplement 1. Trial protocol and statistical analysis plan

Supplement 2. eMethods. eResults. eFigure 1. Simulator components eFigure 2. Simulator configurations eTable 1. Sociodemographic survey of students population eTable 2. Sociodemographic survey of health professionals population eTable 3. Mixed-effect regression model for the mean pressure at each trial for each groups compared with the reference trial (trial 1) during the hands-on training of students group eTable 4. Mixed-effect regression model for the coefficient of variation of pressure at each trial for each groups compared with the reference trial (trial 1) during the hands-on training of students group eTable 5. Mixed-effect regression model for the mean breaths per minute (BPM) at each trial for each groups compared with the reference trial (trial 1) during the hands-on training of students group eTable 6. Mixed-effect regression model for the coefficient of variation of BPM at each trial for each groups compared with the reference trial (trial 1) during the hands-on training of students group eTable 7. Mixed-effect regression model for the mean pressure at each trial for each groups compared with the reference trial (trial 1) during the hands-on training of health professionals group eTable 8. Mixed-effect regression model for the coefficient of variation of pressure at each trial for each groups compared with the reference trial (trial 1) during the hands-on training of health professionals group eTable 9. Mixed-effect regression model for the mean breaths per minute (BPM) at each trial for each groups compared with the reference trial (trial 1) during the hands-on training of health professionals group eTable 10. Mixed-effect regression model for the coefficient of variation of BPM at each trial for each groups compared with the reference trial (trial 1) during the hands-on training of health professionals group eTable 11. Students group final evaluation metrics eTable 12. Health professionals group final evaluation eTable 13. Post hoc Bonferroni test for BPM eTable 14. Pretraining sociodemographic survey eTable 15. Pre- and posttraining knowledge assessment eTable 16. Tutor quantitative evaluation sheet eTable 17. Posttraining TAM survey eTable 18. Structural model results for the PLS-SEM of the TAM survey

Supplement 3. Data Sharing Statement
