# Supplementary material for: Neonatal Ventilation Training Using a Low-Cost, High-Fidelity Simulator in a Low-Resource Setting: A Randomized Clinical Trial
Source: JAMA Netw Open. 2026 Sep 14;9(9):e2633261. doi: 10.1001/jamanetworkopen.2026.33261 (PMC13576422; doi:10.1001/jamanetworkopen.2026.33261)
Supplement: Supplementary file 1 [file jamanetwopen-e2633261-s001.pdf]

Study Protocol and Statistical Analysis Plan

Experimental Study and Training for Tanzanian Students and Health Professionals With a New Simulator for Manual Neonatal Ventilation

ClinicalTrials.gov ID NCT07665164

Link: https://clinicaltrials.gov/study/NCT07665164

Sponsor Scuola Superiore di Studi Universitari e di Perfezionamento Sant'Anna

Information provided by Arianna Menciassi, Scuola Superiore di Studi Universitari e di Perfezionamento Sant'Anna (Responsible Party)

Last Update Posted 2026-06-24

Study Overview

Brief Summary

The goal of this clinical trial is to build capacity and assess the teaching effectiveness of a new simulator for manual neonatal ventilation in third-year nursing and clinical officer students, as well as healthcare professionals from the neonatology, gynecology, and pediatric departments.

The main questions it aims to answer are:

- Does the high-fidelity active setup of the simulator improve the percentage of target pressure achieved and the mean ventilation frequency compared to other configurations?
- How do different simulator configurations affect the training outcomes of unexperienced and experienced personnel, based on objective evaluations by expert clinicians and knowledge surveys?
- What is the self-reported usability and perceived usefulness of the different simulator configurations among the trainees?

Researchers will compare a medium-fidelity passive setup, a high-fidelity passive setup with mechanical feedback, and a high-fidelity active setup with both mechanical and sensor feedback to see if the high-fidelity active configuration results in significantly higher clinical performance scores and better achievement of ventilation targets.

Participants will:

- Attend theoretical frontal lectures covering manual emergency ventilation procedures and medical simulation.
- Complete pre-training and post-training surveys to assess baseline knowledge, evaluate acquired skills, and report on the simulator's usability.
- Participate in hands-on simulator training, practicing the clinical procedure (positioning the newborn, positioning the mask, and ventilating) three times.
- Undergo a final simulation session for evaluation, where performance is objectively scored by a tutor and automatically recorded by the simulator's sensors.

Detailed Description

Background and Rationale

Neonatal mortality in sub-Saharan Africa remains disproportionately high-26 deaths per 1 ,000 live births in 2023, which is 8.7 times higher than rates in Europe and North America. A significant portion of these deaths are attributable to preventable and treatable conditions occurring during birth. While skilled birth attendance can prevent up to 40% of neonatal deaths, assistance rates in sub-Saharan Africa are drastically low (40%) compared to high-income countries (90%).

Consequently, strengthening the capacity of the health workforce to deliver essential newborn care is a critical priority.

Neonatal resuscitation is a complex, time-sensitive procedure. Due to severe limitations in access to advanced respiratory technologies in these regions, manual ventilation using a self-inflating bag and face mask constitutes the primary method of respiratory support. Because manual ventilation is highly operator-dependent and can cause substantial harm if performed incorrectly, technical proficiency is paramount. Simulation-based training is internationally recommended to improve provider competence, yet data on the real-world implementation, acceptability, and educational effectiveness of low-cost, high-fidelity simulators tailored to low-resource settings remains limited. This study evaluates a newly designed simulator developed specifically for the Tanzanian environment to ensure healthcare workers can acquire and maintain resuscitation skills.

Study Objectives

The specific objectives of this study are:

1. to evaluate the educational effectiveness of different simulator configurations across participants with varying levels of clinical experience, using objective performance metrics collected by the simulator.
2. to assess the acquisition of core manual ventilation competencies among inexperienced medical students through objective tutor evaluation and knowledge assessment.
3. to assess skill reinforcement among experienced clinicians through objective tutor evaluation and knowledge assessment.
4. to examine user-reported usability and perceived usefulness of the simulator configurations.

Investigational Device Details

The novel, low-cost, high-fidelity neonatal simulator is designed to replicate neonatal airway anatomy and ventilation mechanics while providing objective performance feedback.

Anatomical Construction: The simulator integrates a physical manikin with internal anatomy designed to mimic the upper airways, lungs, and stomach.

Physiological Mechanics: The device mimics physiological movements, specifically the visible rise of the chest and abdomen during successful ventilation. It also features a mechanical obstruction of the esophagus if the head is not correctly placed in the required "sniffing" position.

Sensory and Digital Interface: The system incorporates pressure sensors and connects to a mobile user interface (app) that enables real-time data acquisition. The app provides the operator with real-time visual and auditory feedback regarding ventilation rate and pressure.

Study Design and Allocation

This is a prospective, randomized, and controlled study conducted at the Tosamaganga Regional Referral Hospital (TRRH) and the Tosamaganga Institute of Health and Allied Sciences (TIHAS) in the Iringa region of Tanzania.

The study population consists of two cohorts: second-year undergraduate clinical officer students at TIHAS (with no prior manual ventilation experience) and local health professionals (nursing, midwifery, and neonatal care) working at TRRH. A total of 42 students and 37 health professionals were enrolled.

Participants within each cohort were randomly assigned to one of three groups (through a computer-based random allocation process) to evaluate the teaching effectiveness of different simulator configurations:

- Group A (Medium-fidelity passive setup): (14 students, 12 professionals). The simulator provides no mechanical feedback (chest rise) and no mobile user interface guidance.
- Group B (High-fidelity passive setup): (14 students, 13 professionals). The simulator provides mechanical feedback via chest and/or abdominal rise, but no mobile user interface guidance.
- Group C (High-fidelity active setup): (14 students, 12 professionals). The simulator provides full mechanical feedback alongside real-time sensor-based guidance via the mobile user interface (visual and auditory feedback).

Study Protocol and Workflow

The training course was delivered over four consecutive days (June 17-20, 2025).

- Day 1 (Theoretical Phase):

  Participants completed a pre-training survey to assess baseline knowledge and sociodemographic characteristics. This was followed by half-day standardized frontal lectures on manual emergency ventilation and the principles of medical simulation.

- Days 2 and 3 (Hands-on Training):

  Participants engaged in two hands-on sessions (one per day with a minimum 24-hour washout period). After a clinical demonstration and a 1 -minute familiarization period, participants performed three 1 -minute ventilation trials per session (six trials total per participant) according to their randomized group configuration. Each trial involved newborn positioning, mask placement, and target ventilation delivery.

- Day 4 (Evaluation Phase):

  All participants completed a standardized evaluation session using Configuration B (mimicking real clinical conditions with only chest rise feedback). Participants performed a single 1 -minute ventilation trial. Performance was objectively assessed by a tutor and via simulator-derived data. Participants subsequently completed two post-training surveys assessing acquired knowledge and simulator acceptability.

Data Collection and Statistical Analysis Plan

All data were anonymized using unique numeric identifiers to link datasets across sessions. Data triangulation was achieved through three primary streams: automated simulator data, expert tutor evaluations, and self-reported surveys.

- Primary Outcome Analysis (Educational Effectiveness): The primary outcome was ventilatory performance measured by the simulator during the six training trials, specifically the mean and standard deviation (Coefficient of Variation) of the ventilation rate (target: 40 BPM) and peak inspiratory pressure (target: 25 cmH2O). Longitudinal evolution across the trials was analyzed using linear mixed-effects models, evaluating trial number, group assignment, and their interaction.

- Secondary Outcome Analysis (Knowledge and Skill acquisition/Reinforcement): Assessed via data from the final Day 4 evaluation session. Outcomes included simulator data, structured tutor performance scores (0 = poor, 0.5 = average, 1 = good), and theoretical knowledge scores (pre/post-training questionnaire). Continuous variables were analyzed using ANOVA (ANalysis Of VAriance) a (following Shapiro-Wilk testing for normality), and paired theoretical knowledge was analyzed using the Wilcoxon test.

- Tertiary Outcome Analysis (Simulator Acceptability): User-reported acceptability was measured across four Technology Acceptance Model (TAM) dimensions: Perceived Usefulness (PU), Perceived Ease-of-Use (PEU), Behavioral Intention (BI), and Fidelity of the Simulator (FS), rated on a 7-point Likert scale. Associations between these latent variables and proposed cause-effect hypotheses were tested using a partial least squares structural equation model.

Official Title

Experimental Study and Training for Tanzanian Students and Health Professionals With a New Simulator for Manual Neonatal Ventilation

Conditions

Teaching Manual Neonatal Ventilation

Intervention/Treatment

Other: Training

Other Study ID Numbers

NIMR/HQ/R.8a/Vol.IX/4983

Study Start (Actual)

2025-06-1 7

Primary Completion (Actual)

2025-06-20

Study Completion (Actual)

2025-06-20

Enrollment (Actual)

79

Study Type

Interventional

Contacts and Locations

Location: Tanzania, Iringa

Tosamaganga Regional Referral Hospital

Tosamaganga, Iringa, Tanzania, P.O. Box 1 1

Participation Criteria

Eligibility Criteria

Description

Inclusion Criteria:

- Third-year nursing students enrolled at the Tosamaganga Institute of Health and Allied Sciences (TIHAS).
- Third-year clinical officer students enrolled at TIHAS.
- Healthcare professionals working at Tosamaganga Hospital within the neonatology, gynecology, midwifery, and pediatrics departments.
- Individuals who voluntarily choose to attend the course.

Exclusion Criteria:

- Healthcare professionals working in fields outside of nursing, gynecology, midwifery, neonatology, and pediatrics.
- Participants who fail to complete the full training program, either by not attending all sessions or by prematurely withdrawing from the course.

Ages Eligible for Study

Adult, Older Adult

Sexes Eligible for Study

All

Accepts Healthy Volunteers

Yes

Study Plan

Design Details

Primary Purpose: Health Services Research
Allocation: Randomized. Researchers provided the identification numbers to the participants.
Interventional Model: Parallel Assignment
Masking: Double (Investigator, Outcomes Assessor). The evaluator for the final assessment did not know the participants. Data for analysis were anonymized and copied in datasets. Outcome assessor was not aware of the intervention given to the participants.

Arms and Interventions

Participant Group/Arm	Intervention/Treatment
Experimental:<br><br>Training with simulator configuration A<br><br>Group A used a medium-fidelity passive setup of the simulator without mechanical feedback or mobile user interface guidance.	Other: Training<br><br>• Medical training on manual neonatal ventilation with physical simulator
Experimental:<br><br>Training with simulator configuration B<br><br>Group B used a high-fidelity passive setup of the simulator providing mechanical feedback through chest and/or abdominal rise, without mobile user interface guidance.	Other: Training<br><br>• Medical training on manual neonatal ventilation with physical simulator
Experimental:<br><br>Training with simulator configuration C<br><br>Group C used a high-fidelity active setup of the simulator combining mechanical feedback with real-time sensor-based guidance via a mobile user interface.	Other: Training<br><br>• Medical training on manual neonatal ventilation with physical simulator

Primary Outcome Measures

Outcome Measure	Measure Description	Time Frame
Peak Inspiratory Pressure (PIP) (Objective 1)	The primary outcome, used to assess the Objective 1 , was the ventilatory performance measured by the simulator, i.e. Peak Inspiratory Pressure (PIP) during hands-on training on Day 2 and Day 3, and the evaluation session on Day 4. In accordance with international guidelines, we considered as gold-standard a PIP of 25 cmH_2O (centimeters of water). For each participant and trial, we computed the mean and standard deviation of PIP.	Day 2, Day 3 and Day 4
Ventilation Rate (Objective 1)	The other primary outcome, used to assess the Objective 1 , was the ventilatory performance measured by the simulator, i.e. Ventilation Rate during hands-on training on Day 2 and Day 3, and the evaluation session on Day 4. In accordance with international guidelines, we considered as gold-standard a Ventilation Rate of 40 breaths per minute. For each participant and trial, we computed the mean and standard deviation of the Ventilation Rate .	Day 2, Day 3 and Day 4

Secondary Outcome Measures

Outcome Measure	Measure Description	Time Frame
Skill acquisition assessment (Objectives 2 and 3)	The secondary outcomes, used to evaluate Objectives 2 and 3, were acquisition and reinforcement of manual ventilation knowledge and skills, assessed through: (1) data from the simulator sensors acquired during the final evaluation session, (2) objective tutor-based performance evaluation using a structured quantitative scoring sheet on a three-point scale (0 = poor, 0.5 = average, 1 = good), (3) theoretical knowledge assessment (pre and post-training true/false questionnaire).	Day 1 and day 4

Other Outcome Measures

Outcome Measure	Measure Description	Time Frame
User-reported usability and perceived usefulness (Objective 4)	The tertiary outcome used to evaluate Objective 4, were user-reported measures of simulator acceptability over the four dimensions of the TAM scale (Technology Acceptance Model): Perceived Usefulness (PU), Perceived Ease-of-Use (PEU), Behavioural Intention (BI), and Fidelity of the Simulator (FS). The items were tailored to the characteristics of the simulator and rated on 7 points Likert scales. A final aggregated score was obtained.	Day 4

Collaborators and Investigators

Sponsor

Scuola Superiore di Studi Universitari e di Perfezionamento Sant'Anna

Collaborators

- Doctors with Africa - CUAMM Tosamaganga
- Regional Referral Hospital
- National Institute for Medical Research, Tanzania

Publications

Maglio S, Tamirat S, Tesfaye M, Wolde M, Tognarelli S, Menciassi A, Facci E. Toward the design of a tailored training course for birth assistance: an Ethiopian experience. Pan Afr Med J. 2024 Jan 26;47:32. (https://pubmed.ncbi.nlm.nih.gov/38586071)

Turatsinze S, Willson A, Sessions H, Cartledge PT. Medical student satisfaction and confidence in simulation-based learning in Rwanda - Pre and post-simulation survey research. Afr J Emerg Med. 2020. (https://pubmed.ncbi.nlm.nih.gov/3261 291 4)

Boling B, Hardin-Pierce M. The effect of high-fidelity simulation on knowledge and confidence in critical care training: An integrative review. Nurse Educ Pract. 201 6 Jan;1 6(1):287-93. (https://pubmed.ncbi.nlm.nih.gov/2652021 3)

Lee HC, Strand ML, Finan E, Illuzzi J, Kamath-Rayne BD, Kapadia V, Mahgoub M, Niermeyer S, Schexnayder SM, Schmolzer GM, Weglarz J, Williams AL, Weiner GM, Wyckoff M, Yamada NK, Szyld E. Part 5: Neonatal Resuscitation: 2025 American Heart Association and American Academy of Pediatrics Guidelines for Cardiopulmonary Resuscitation and Emergency Cardiovascular Care. Circulation. 2025 (https://pubmed.ncbi.nlm.nih.gov/41 1 22887)

More Information

Plan for Individual Participant Data (IPD)

Plan to Share Individual Participant Data (IPD)?

Yes

IPD Plan Description

Anonymized and aggregated data corresponding to the primary outcomes (mean and standard deviations of PIP and Ventilation Rate per trial per group).

IPD Sharing Access Criteria

Data can be accessed by reseachers upon request only for reseach purpose and with a signed data access agreement after approval of a proposal. Any request can be sent to arianna.menciassi@santannapisa.it

IPD Sharing Time Frame

Starting date: Janualy 1 st 2026 End date: November 30st 2026
