# Supplementary material for: Neonatal Ventilation Training Using a Low-Cost, High-Fidelity Simulator in a Low-Resource Setting: A Randomized Clinical Trial
Source: JAMA Netw Open. 2026 Sep 14;9(9):e2633261. doi: 10.1001/jamanetworkopen.2026.33261 (PMC13576422; doi:10.1001/jamanetworkopen.2026.33261)
Supplement: Supplementary file 2 [file jamanetwopen-e2633261-s002.pdf]

Supplemental Online Content

Mocellin D, Maglio S, Nanyaro M, et al. Neonatal ventilation training using a low-cost high-fidelity simulator in a low-resource setting. *JAMA Netw Open.* 2026;9(9):e2633261. doi:10.1001/jamanetworkopen.2026.33261

eMethods.

eResults.

eFigure 1. Simulator components

eFigure 2. Simulator configurations

eTable 1. Sociodemographic survey of students population

eTable 2. Sociodemographic survey of health professionals population

eTable 3. Mixed-effect regression model for the mean pressure at each trial for each groups compared with the reference trial (trial 1) during the hands-on training of students group

eTable 4. Mixed-effect regression model for the coefficient of variation of pressure at each trial for each groups compared with the reference trial (trial 1) during the hands-on training of students group

eTable 5. Mixed-effect regression model for the mean breaths per minute (BPM) at each trial for each groups compared with the reference trial (trial 1) during the hands-on training of students group

eTable 6. Mixed-effect regression model for the coefficient of variation of BPM at each trial for each groups compared with the reference trial (trial 1) during the hands-on training of students group

eTable 7. Mixed-effect regression model for the mean pressure at each trial for each groups compared with the reference trial (trial 1) during the hands-on training of health professionals group

eTable 8. Mixed-effect regression model for the coefficient of variation of pressure at each trial for each groups compared with the reference trial (trial 1) during the hands-on training of health professionals group

eTable 9. Mixed-effect regression model for the mean breaths per minute (BPM) at each trial for each groups compared with the reference trial (trial 1) during the hands-on training of health professionals group

eTable 10. Mixed-effect regression model for the coefficient of variation of BPM at each trial for each groups compared with the reference trial (trial 1) during the hands-on training of health professionals group

eTable 11. Students group final evaluation metrics

© 2026 Mocellin D et al. *JAMA Network Open*.

eTable 12. Health professionals group final evaluation
eTable 13. Post hoc Bonferroni test for BPM
eTable 14. Pretraining sociodemographic survey
eTable 15. Pre- and posttraining knowledge assessment
eTable 16. Tutor quantitative evaluation sheet
eTable 17. Posttraining TAM survey
eTable 18. Structural model results for the PLS-SEM of the TAM survey

This supplemental material has been provided by the authors to give readers additional information about their work.

eMethods.

1. Neonatal ventilation simulator development

The neonatal ventilation simulator was developed at the Surgical Robotics and Allied Technologies Laboratory of the BioRobotics Institute, Scuola Superiore Sant'Anna (SSSA, Italy). The simulator has the aim of providing user-friendly training in manual neonatal mechanical ventilation replicating neonatal anatomy and ventilation mechanics. The simulator integrates the head skeleton of a commercial neonatal ventilation manikin (Newborn Anne, Laerdal, Norway) with a newly designed and 3D-printed body. The upper airways are manufactured with silicone material (Ecoflex 00-30, Smooth-On, USA) and molding technique, while the lungs and stomach are reproduced heat-sealing two PET (Polyethylene terephthalate) sheets to create bags with internal volume close to the neonatal tidal volume. These components are interconnected through tubing with a commercial pressure sensor (ABP2DRRT005PD2A3XX, Honeywell, USA) that acquires lung pressures for evaluating ventilation performance during the simulation. The entire body and face are covered with a silicone-based skin (Ecoflex 00-10) for anatomical realism. The simulator reproduces the key anatomical components involved in ventilation and allows visualization of physiological movements such as chest and abdominal rise (eFigure 1). Additionally, the simulator allows for the positioning of the head in the "sniffing" position to optimize airway patency and minimize esophageal air insufflation [1].

To monitor and provide feedback on ventilation performance, data are collected and processed by an Arduino Nano RP2040® microcontroller (Arduino, Italy) that transmits information via Bluetooth communication to an ad-hoc programmed Android-based application. The application provides an essential mobile user interface (MUI) allowing the operator to initiate the training sessions, monitor real-time ventilation pressure, practice ventilation frequency, and store data for subsequent analysis. The pressure sensor is displayed in real time through a moving green bar, facilitating interpretation by the trainee, while an auditory cue (music set at 40 beats per minute) guides users toward the target ventilation frequency during training.
Supplementary eFigure 2 shows the three configurations of the simulator used in the study. The configuration A features an external rigid cover to hide chest movement. In configuration B the MUI is hidden to the trainee while chest movements are visible. Configuration C provides both mechanical feedback and sensor-based guidance through the mobile GUI, allowing learners to see real-time pressure inside the manikin's lungs and to receive an auditory signal to guide the ventilation rate.
Prior to the training course in Tanzania, SSSA engineers conducted a preliminary evaluation of the simulator with clinical experts of the neonatology department of the Azienda Ospedaliera Nord Ovest Toscana (Italy), assessing the simulator's suitability for neonatal ventilation training.

2. Sample size calculation

An a priori sample size calculation was performed based on the primary outcome of ventilation performance, expressed as breaths per minute with target peak pressure. According to international guidelines, optimal neonatal ventilation corresponds to approximately 40 breaths per minute.
The calculation focused on two training conditions expected to yield the largest performance difference: a high-fidelity active setup providing both mechanical and sensor-based feedback (Group C), and a medium-fidelity passive setup without feedback (Group A). Based on preliminary assumptions, mean ventilation rates of 37 breaths per minute and 30 breaths per minute were expected for the high-fidelity and medium-fidelity groups, respectively.
Assuming a common standard deviation of 5 breaths per minute, the standardized effect size was estimated as 1.4. Using a two-sided α level of 0.05 and a statistical power of 80%, the required sample size was calculated using the formula $n = 2(Z\alpha/2 + Z\beta)\hat{}2)/(effect\ size\hat{}2\ )$, resulting in a minimum of 8 participants per group. To account for potential non-response or incomplete data, a 20% increase was applied, yielding a final target sample size of 10 participants per group.

3. Ethics and Data Protection

The study protocol, including data collection procedures and survey administration, was approved by the National Health Research Ethics Committee of the National Institute for Medical Research (NIMR), Tanzania, with the certificate number NIMR/HQ/R.8a/Vol.IX/4983. All data were de-identified and anonymized prior to analysis. No personal or contact information capable of identifying participants (e.g., name, date of birth, email address) was collected at any stage. Each participant was assigned a unique numeric identification code, randomly selected from a predefined set, which was used to link responses across surveys without revealing identity. Survey data were transcribed electronically into spreadsheets containing only unique numeric participant identifiers. All data were stored on secure internal servers of NIMR and used exclusively for research purposes. A Data Transfer Agreement was established to allow data sharing with Scuola Superiore Sant'Anna.

4. Further statistical considerations

© 2026 Mocellin D et al. *JAMA Network Open*.

To evaluate the longitudinal evolution of ventilation performance across the six training attempts (trials 1–6) (Objective 1), we fitted linear mixed-effects models separately for each outcome: mean peak inspiratory pressure (PIP), ventilation rate (breaths per minute (BPM)), and their corresponding coefficients of variation (CV_{PIP} and CV_{BPM}) computed as the ratio of the standard deviation to the mean. All outcomes were recorded repeatedly for the same participant across trials; therefore, mixed models were preferred over repeated-measures ANOVA (Analysis of Variance) due to their ability to account for within-subject correlation and to handle unbalanced data structures.
For each outcome, we estimated a mixed-effects model of the form:

$$\text{Outcome}_{ij} = \beta_0 + \beta_1(\text{Trial}_j) + \beta_2(\text{Group}_i) + \beta_3(\text{Trial}_j \times \text{Group}_i) + u_i + \varepsilon_{ij},$$

where u_i represents a participant-specific random intercept capturing inter-individual differences at baseline, and ε_{ij} is the residual error. Trial (1–6) and group assignment (A, B, C) were included as categorical fixed effects, together with their interaction term, to examine whether performance trajectories differed between groups. The intercept (β_0) represents the expected value of the outcome at Trial 1 for the reference group. The trial coefficients (β_1) capture the change in the outcome at each subsequent trial compared with Trial 1, reflecting the overall learning or deterioration trend. The group coefficients (β_2) indicate baseline differences between the experimental groups at Trial 1. Finally, the interaction terms (β_3) describe whether the evolution of performance over time differs by group—that is, whether groups show distinct learning trajectories.

© 2026 Mocellin D et al. *JAMA Network Open*.

eResults

Study Populations Characteristics

Students population
We enrolled 42 student participants (median age: 21 years, IQR: 20–22). Females comprised 59.5% of the cohort (n=25). The majority (97.6%, n=41) were single. All 42 participants (100%) were enrolled in the nursing and midwifery training program. Prior hands-on training activities were reported by 66.7% (n=28), while 78.6% (n=33) reported prior health-related work experience. Household economic sustainability was reported as "well" by 61.9% (n=26), "quite well" by 28.6% (n=12), and "not well" by 7.1% (n=3). All students (100%, n=42) expressed interest in participating in the course and reported satisfaction with the training experience. No statistically significant differences were found in baseline characteristics between groups (all p-values > 0.05), confirming successful randomization.

Health professionals population
We enrolled 37 health professionals (median age: 26 years, IQR: 23–29). Females comprised 81.1% of the cohort (n=30). Clinical specialties included midwiferies (78.4%, n=29), nursing (13.5%, n=5), and other specialties (8.1%, n=3). Prior hands-on training activities were reported by 91.9% (n=34), while 94.6% (n=35) reported prior health-related work experience. Household economic sustainability was reported as "well" by 54.1% (n=20), "quite well" by 24.3% (n=9), and "not well" by 16.2% (n=6). All health professionals (100%, n=37) expressed interest in participating in the course. No statistically significant differences were found in baseline characteristics between groups (all p-values > 0.05), confirming successful randomization.

Educational effectiveness of different simulator configurations

These tables show the results of a mixed-effects regression model evaluating changes in the four measured dimensions (mean pressure, coefficient of variation of pressure, mean breaths per minute (BPM) and coefficient of variation of BPM) across repeated trials within each group. For each group (A, B, and C), trial 1 was used as the reference trial. Therefore, the coefficients indicate how much the measured dimension in each subsequent trial differs from trial 1 within the same group. Positive coefficients indicate higher measured dimension compared with trial 1, while negative coefficients indicate lower measured dimension. The p-values < 0.05 show a statistically significant difference, and the 95% confidence intervals indicate the estimated range of each difference. A_n = trial number n of group A, B_n = trial number n of group B, C_n = trial number n of group C with n=1:6.

Final evaluation

Students population
The final evaluation phase assessed objective ventilatory performance in all 42 student participants. Peak inspiratory pressure (PIP) and ventilation rate (breaths per minute (BPM)) were analyzed for each group, along with tutor-assigned performance scores and knowledge evaluation.

Health professionals population
The final evaluation phase assessed objective ventilatory performance in 37 health professionals. Peak inspiratory pressure (PIP) and ventilation rate (breaths per minute (BPM)) were analyzed for each group, along with tutor-assigned performance scores and knowledge evaluation.

© 2026 Mocellin D et al. *JAMA Network Open*.

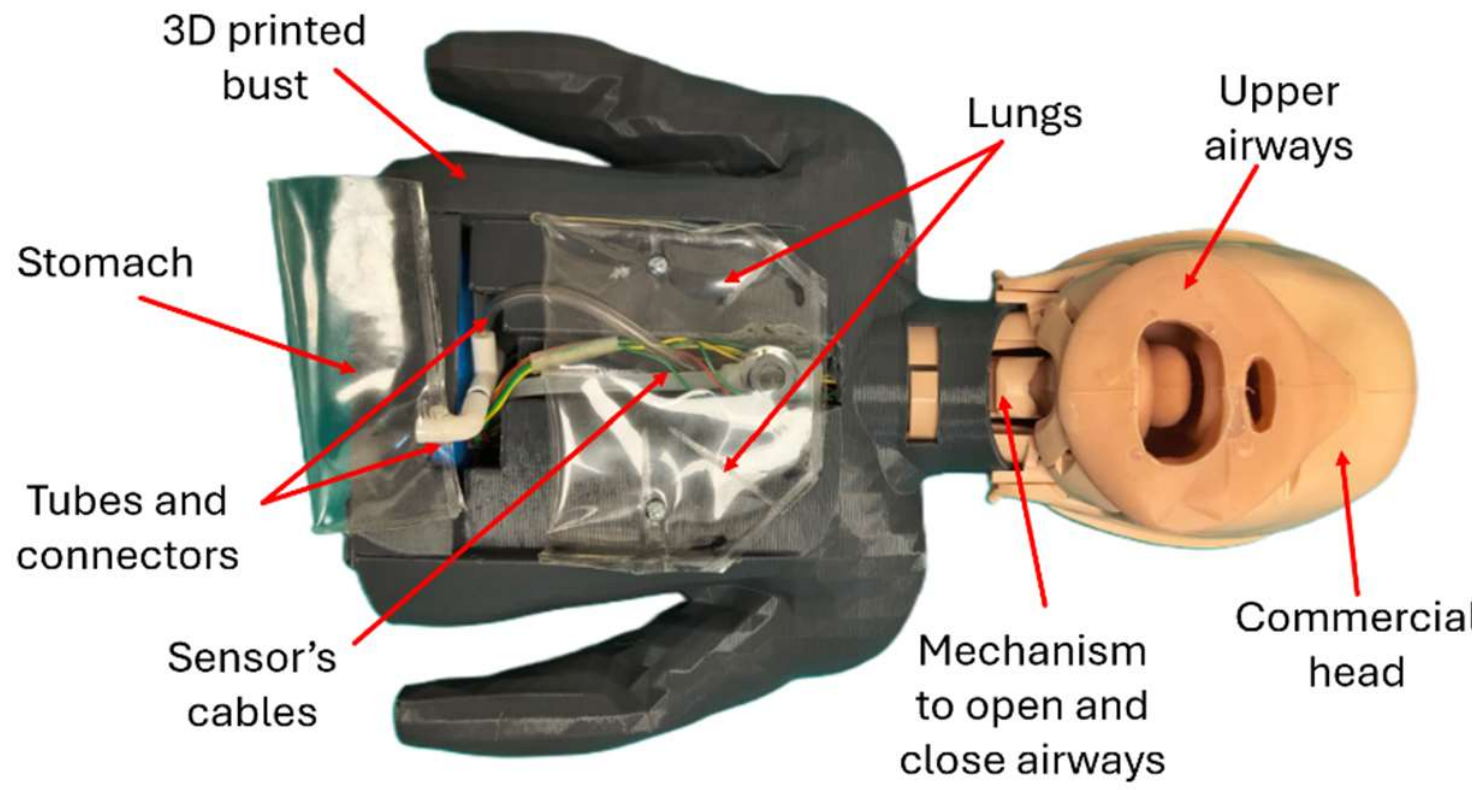

eFigure 1. Simulator components.

© 2026 Mocellin D et al. *JAMA Network Open*.

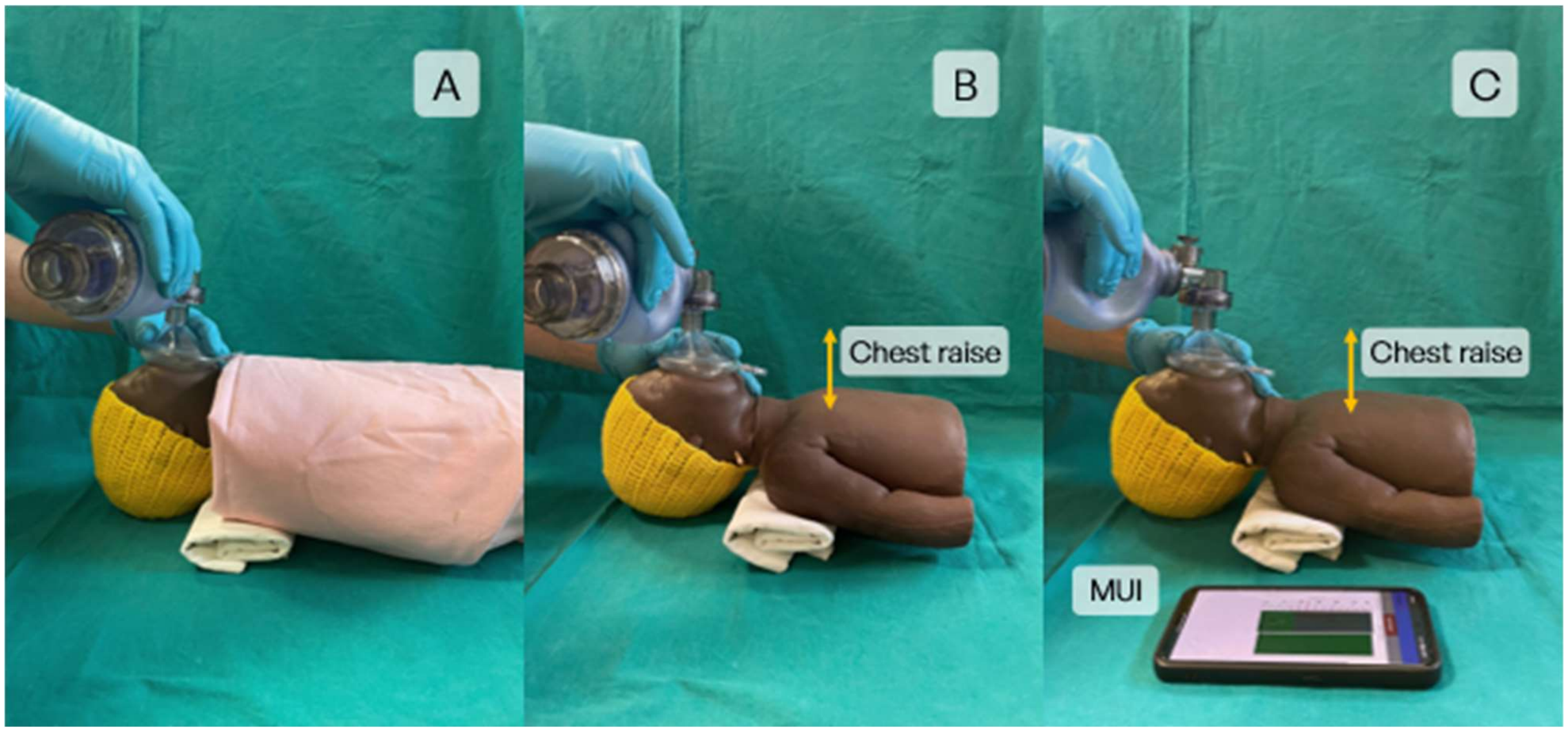

eFigure 2. Simulator configurations: a) medium-fidelity passive setup without any feedback; b) high-fidelity passive setup providing mechanical feedback through chest raise; c) high-fidelity active setup combining mechanical feedback with real-time sensor-based guidance via a mobile user interface (MUI).

© 2026 Mocellin D et al. *JAMA Network Open*.

eTable 1. Sociodemographic survey of students population

	Total	Group A	Group B	Group C	p-value
N	42	14	14	14	
Age__n____, median (IQR)	21.0 (20.0, 22.0)	21.0 (20.0, 22.0)	22.0 (20.0, 24.0)	20.0 (19.0, 21.0)	0.073
Sex__M__F____					0.15
F	25 (59.5%)	8 (57.1%)	6 (42.9%)	11 (78.6%)	
M	17 (40.5%)	6 (42.9%)	8 (57.1%)	3 (21.4%)	
Civil_status__S__M____					0.36
M	1 (2.4%)	0 (0.0%)	1 (7.1%)	0 (0.0%)	
S	41 (97.6%)	14 (100.0%)	13 (92.9%)	14 (100.0%)	
Specializzation__Midw__Nurs__Oth					
CO	42 (100.0%)	14 (100.0%)	14 (100.0%)	14 (100.0%)	
Prior_hands_on_training_activiti					0.088
/	3 (7.1%)	2 (14.3%)	1 (7.1%)	0 (0.0%)	
N	11 (26.2%)	6 (42.9%)	4 (28.6%)	1 (7.1%)	
Y	28 (66.7%)	6 (42.9%)	9 (64.3%)	13 (92.9%)	
Household_economic_sustainabilit					0.47
/	1 (2.4%)	0 (0.0%)	1 (7.1%)	0 (0.0%)	
Not well	3 (7.1%)	1 (7.1%)	2 (14.3%)	0 (0.0%)	
Quite well	12 (28.6%)	5 (35.7%)	2 (14.3%)	5 (35.7%)	
Well	26 (61.9%)	8 (57.1%)	9 (64.3%)	9 (64.3%)	
Prior_work_studies_experience__Y					0.32
/	1 (2.4%)	0 (0.0%)	1 (7.1%)	0 (0.0%)	
N	5 (11.9%)	3 (21.4%)	2 (14.3%)	0 (0.0%)	
Yhealth	33 (78.6%)	10 (71.4%)	11 (78.6%)	12 (85.7%)	
Ynot	3 (7.1%)	1 (7.1%)	0 (0.0%)	2 (14.3%)	
Interest_in_partecipating__Y__N_					
Y	42 (100.0%)	14 (100.0%)	14 (100.0%)	14 (100.0%)	
Satisfaction_about_the_frontal_l, median (IQR)	3.0 (3.0, 3.0)	3.0 (3.0, 3.0)	3.0 (3.0, 3.0)	3.0 (3.0, 3.0)	0.37
Overall_satisfaction_about_the_t, median (IQR)	5.0 (5.0, 5.0)	5.0 (5.0, 5.0)	5.0 (4.0, 5.0)	5.0 (4.0, 5.0)	0.28
Satisfaction_with_the_performanc, median (IQR)	5.0 (5.0, 5.0)	5.0 (5.0, 5.0)	5.0 (5.0, 5.0)	5.0 (4.0, 5.0)	0.59

© 2026 Mocellin D et al. *JAMA Network Open*.

eTable 2. Sociodemographic survey of health professionals population

Factor	Value	Group A	Group B	Group C	p-value
N	37	12	13	12	
Age__n____, median (IQR)	26.0 (23.0, 29.0)	24.0 (22.5, 26.5)	28.0 (24.0, 34.0)	26.0 (23.5, 29.0)	0.14
Sex__M__F____					0.52
F	30 (81.1%)	11 (91.7%)	10 (76.9%)	9 (75.0%)	
M	7 (18.9%)	1 (8.3%)	3 (23.1%)	3 (25.0%)	
Civil_status__S__M____					0.25
M	11 (29.7%)	2 (16.7%)	6 (46.2%)	3 (25.0%)	
S	26 (70.3%)	10 (83.3%)	7 (53.8%)	9 (75.0%)	
Specializzation__Midw__Nurs__Oth					0.053
Mi	29 (78.4%)	11 (91.7%)	12 (92.3%)	6 (50.0%)	
Nu	5 (13.5%)	1 (8.3%)	0 (0.0%)	4 (33.3%)	
Ot	3 (8.1%)	0 (0.0%)	1 (7.7%)	2 (16.7%)	
Prior_hands_on_training_activiti					0.57
/	1 (2.7%)	0 (0.0%)	1 (7.7%)	0 (0.0%)	
N	2 (5.4%)	1 (8.3%)	0 (0.0%)	1 (8.3%)	
Y	34 (91.9%)	11 (91.7%)	12 (92.3%)	11 (91.7%)	
Household_economic_sustainabilit					0.27
/	2 (5.4%)	0 (0.0%)	2 (15.4%)	0 (0.0%)	
Not well	6 (16.2%)	3 (25.0%)	1 (7.7%)	2 (16.7%)	
Quite well	9 (24.3%)	2 (16.7%)	2 (15.4%)	5 (41.7%)	
Well	20 (54.1%)	7 (58.3%)	8 (61.5%)	5 (41.7%)	
Prior_work_studies_experience__Y					0.41
N	1 (2.7%)	0 (0.0%)	1 (7.7%)	0 (0.0%)	
Yhealth	35 (94.6%)	11 (91.7%)	12 (92.3%)	12 (100.0%)	
Ynot	1 (2.7%)	1 (8.3%)	0 (0.0%)	0 (0.0%)	
Interest_in_partecipating__Y__N_					
Y	37 (100.0%)	12 (100.0%)	13 (100.0%)	12 (100.0%)	
Satisfaction_about_the_frontal_l, median (IQR)	3.0 (3.0, 3.0)	3.0 (3.0, 3.0)	3.0 (3.0, 3.0)	3.0 (2.5, 3.0)	0.13
Overall_satisfaction_about_the_t, median (IQR)	5.0 (5.0, 5.0)	5.0 (5.0, 5.0)	5.0 (4.0, 5.0)	5.0 (5.0, 5.0)	0.37
Satisfaction_with_the_performanc, median (IQR)	5.0 (4.0, 5.0)	5.0 (4.5, 5.0)	5.0 (4.0, 5.0)	5.0 (4.0, 5.0)	0.72

© 2026 Mocellin D et al. *JAMA Network Open*.

eTable 3. Mixed-effect regression model for the mean pressure at each trial for each groups compared with the reference trial (trial 1) during the hands-on training of students group.

Mean pressure	Coefficient	P>|z|	[95% confidence interval]	
A_2 vs A_1	-1,126159	0,194	-2,824638	0,57232
A_3 vs A_1	-0,642197	0,459	-2,340676	1,056282
A_4 vs A_1	-1,292502	0,144	-3,026745	0,441742
A_5 vs A_1	-0,4621567	0,594	-2,160636	1,236322
A_6 vs A_1	-0,8243987	0,341	-2,522878	0,87408
B_2 vs B_1	2,385341	0,007	0,6598985	4,110784
B_3 vs B_1	2,247182	0,01	0,5480731	3,946292
B_4 vs B_1	2,907437	0,001	1,17247	4,642405
B_5 vs B_1	2,504235	0,004	0,8051258	4,203344
B_6 vs B_1	2,656213	0,003	0,9212457	4,39118
C_2 vs C_1	0,3175823	0,708	-1,345096	1,98026
C_3 vs C_1	0,3610329	0,67	-1,301645	2,023711
C_4 vs C_1	-0,2471397	0,771	-1,909818	1,415538
C_5 vs C_1	1,500663	0,077	-0,1620153	3,163341
C_6 vs C_1	0,8688824	0,306	-0,7937957	2,53156

© 2026 Mocellin D et al. *JAMA Network Open*.

eTable 4. Mixed-effect regression model for the coefficient of variation of pressure at each trial for each groups compared with the reference trial (trial 1) during the hands-on training of students group.

CV pressure	Coefficient	P>|z|	[95% confidence interval]	
A_2 vs A_1	-0,037	0,065	-0,07626	0,002271
A_3 vs A_1	-0,03341	0,095	-0,07268	0,005857
A_4 vs A_1	-0,03179	0,12	-0,07189	0,008308
A_5 vs A_1	-0,02946	0,141	-0,06872	0,009808
A_6 vs A_1	-0,03749	0,061	-0,07676	0,001773
B_2 vs B_1	-0,04656	0,022	-0,08644	-0,00667
B_3 vs B_1	-0,04531	0,024	-0,08459	-0,00603
B_4 vs B_1	-0,049	0,017	-0,08911	-0,00888
B_5 vs B_1	-0,04535	0,024	-0,08463	-0,00607
B_6 vs B_1	-0,03867	0,059	-0,07878	0,001449
C_2 vs C_1	-0,02106	0,283	-0,05949	0,017379
C_3 vs C_1	-0,0185	0,346	-0,05693	0,019937
C_4 vs C_1	0,073552	0	0,035118	0,111987
C_5 vs C_1	-0,00277	0,888	-0,0412	0,035666
C_6 vs C_1	0,016791	0,392	-0,02164	0,055226

© 2026 Mocellin D et al. *JAMA Network Open*.

eTable 5. Mixed-effect regression model for the mean breaths per minute (BPM) at each trial for each groups compared with the reference trial (trial 1) during the hands-on training of students group.

Mean BPM	Coefficient	P>|z|	[95% confidence interval]	
A_2 vs A_1	0,889524	0,261	-0,66256	2,441603
A_3 vs A_1	2,810054	0	1,257974	4,362134
A_4 vs A_1	3,80397	0	2,217721	5,390219
A_5 vs A_1	4,261074	0	2,708995	5,813154
A_6 vs A_1	3,892192	0	2,340112	5,444272
B_2 vs B_1	-1,07906	0,179	-2,65445	0,496326
B_3 vs B_1	-0,2193	0,782	-1,77248	1,333886
B_4 vs B_1	-0,57957	0,474	-2,1671	1,007951
B_5 vs B_1	-0,89973	0,256	-2,45291	0,653456
B_6 vs B_1	0,110508	0,891	-1,47702	1,698032
C_2 vs C_1	1,08037	0,163	-0,43771	2,598451
C_3 vs C_1	1,062583	0,17	-0,4555	2,580664
C_4 vs C_1	0,908691	0,241	-0,60939	2,426771
C_5 vs C_1	0,934154	0,228	-0,58393	2,452234
C_6 vs C_1	0,637762	0,41	-0,88032	2,155842

© 2026 Mocellin D et al. *JAMA Network Open*.

eTable 6. Mixed-effect regression model for the coefficient of variation of BPM at each trial for each groups compared with the reference trial (trial 1) during the hands-on training of students group.

CV of BPM	Coefficient	P>|z|	[95% confidence interval]	
A_2 vs A_1	0,002174	0,819	-0,01642	0,020764
A_3 vs A_1	0,002672	0,778	-0,01592	0,021261
A_4 vs A_1	0,015002	0,121	-0,00398	0,033987
A_5 vs A_1	-0,00012	0,99	-0,01871	0,018468
A_6 vs A_1	-0,01074	0,258	-0,02933	0,007851
B_2 vs B_1	-0,01406	0,145	-0,03294	0,004824
B_3 vs B_1	0,003664	0,699	-0,01493	0,022261
B_4 vs B_1	-0,02443	0,012	-0,04342	-0,00544
B_5 vs B_1	-0,0271	0,004	-0,0457	-0,0085
B_6 vs B_1	-0,02875	0,003	-0,04774	-0,00976
C_2 vs C_1	0,003298	0,722	-0,0149	0,021492
C_3 vs C_1	-0,00987	0,288	-0,02806	0,008329
C_4 vs C_1	0,002775	0,765	-0,01542	0,020969
C_5 vs C_1	-0,00611	0,51	-0,02431	0,012082
C_6 vs C_1	-0,01269	0,172	-0,03089	0,005501

© 2026 Mocellin D et al. *JAMA Network Open*.

eTable 7. Mixed-effect regression model for the mean pressure at each trial for each groups compared with the reference trial (trial 1) during the hands-on training of health professionals group.

Mean pressure	Coefficient	P>|z|	[95% confidence interval]	
A_2 vs A_1	-1,126159	0,194	-2,824638	0,57232
A_3 vs A_1	-0,642197	0,459	-2,340676	1,056282
A_4 vs A_1	-1,292502	0,144	-3,026745	0,441742
A_5 vs A_1	-0,4621567	0,594	-2,160636	1,236322
A_6 vs A_1	-0,8243987	0,341	-2,522878	0,87408
B_2 vs B_1	2,385341	0,007	0,6598985	4,110784
B_3 vs B_1	2,247182	0,01	0,5480731	3,946292
B_4 vs B_1	2,907437	0,001	1,17247	4,642405
B_5 vs B_1	2,504235	0,004	0,8051258	4,203344
B_6 vs B_1	2,656213	0,003	0,9212457	4,39118
C_2 vs C_1	0,3175823	0,708	-1,345096	1,98026
C_3 vs C_1	0,3610329	0,67	-1,301645	2,023711
C_4 vs C_1	-0,2471397	0,771	-1,909818	1,415538
C_5 vs C_1	1,500663	0,077	-0,1620153	3,163341
C_6 vs C_1	0,8688824	0,306	-0,7937957	2,53156

© 2026 Mocellin D et al. *JAMA Network Open*.

eTable 8. Mixed-effect regression model for the coefficient of variation of pressure at each trial for each groups compared with the reference trial (trial 1) during the hands-on training of health professionals group.

CV pressure	Coefficient	P>|z|	[95% confidence interval]	
A_2 vs A_1	0,024211	0,092	-0,00399	0,052417
A_3 vs A_1	0,001748	0,903	-0,02646	0,029954
A_4 vs A_1	-0,01304	0,365	-0,04125	0,015162
A_5 vs A_1	-0,01513	0,293	-0,04333	0,013078
A_6 vs A_1	-0,00692	0,631	-0,03512	0,02129
B_2 vs B_1	-0,04692	0,001	-0,07465	-0,01918
B_3 vs B_1	-0,02387	0,092	-0,0516	0,003868
B_4 vs B_1	-0,03412	0,016	-0,06185	-0,00638
B_5 vs B_1	-0,04313	0,003	-0,0715	-0,01476
B_6 vs B_1	-0,04512	0,001	-0,07286	-0,01739
C_2 vs C_1	-0,01548	0,282	-0,04368	0,012728
C_3 vs C_1	-0,01942	0,177	-0,04763	0,008783
C_4 vs C_1	-0,02862	0,047	-0,05683	-0,00042
C_5 vs C_1	-0,01872	0,193	-0,04693	0,009482
C_6 vs C_1	-0,03215	0,025	-0,06036	-0,00395

© 2026 Mocellin D et al. *JAMA Network Open*.

eTable 9. Mixed-effect regression model for the mean breaths per minute (BPM) at each trial for each groups compared with the reference trial (trial 1) during the hands-on training of health professionals group.

Mean BPM	Coefficient	P>|z|	[95% confidence interval]	
A_2 vs A_1	-0,06389	0,934	-1,56706	1,439288
A_3 vs A_1	0,845817	0,27	-0,65736	2,348993
A_4 vs A_1	-0,64219	0,402	-2,14537	0,860984
A_5 vs A_1	-0,3231	0,674	-1,82628	1,180077
A_6 vs A_1	-0,86224	0,261	-2,36542	0,640937
B_2 vs B_1	0,219361	0,771	-1,25971	1,698429
B_3 vs B_1	1,067906	0,157	-0,41116	2,546974
B_4 vs B_1	-0,06102	0,936	-1,54009	1,418046
B_5 vs B_1	0,773163	0,317	-0,74093	2,28726
B_6 vs B_1	0,399209	0,597	-1,07986	1,878278
C_2 vs C_1	0,057843	0,94	-1,44533	1,561019
C_3 vs C_1	0,454984	0,553	-1,04819	1,958161
C_4 vs C_1	0,779663	0,309	-0,72351	2,282839
C_5 vs C_1	0,530757	0,489	-0,97242	2,033933
C_6 vs C_1	0,86652	0,259	-0,63666	2,369696

© 2026 Mocellin D et al. *JAMA Network Open*.

eTable 10. Mixed-effect regression model for the coefficient of variation of BPM at each trial for each groups compared with the reference trial (trial 1) during the hands-on training of health professionals group.

CV of BPM	Coefficient	P>|z|	[95% confidence interval]	
A_2 vs A_1	-0,00417	0,684	-0,02423	0,0159
A_3 vs A_1	-0,00391	0,703	-0,02398	0,016156
A_4 vs A_1	-0,00528	0,606	-0,02535	0,014781
A_5 vs A_1	-0,01617	0,114	-0,03624	0,003892
A_6 vs A_1	-0,01756	0,086	-0,03762	0,002511
B_2 vs B_1	-0,0169	0,093	-0,03663	0,002831
B_3 vs B_1	-0,02106	0,036	-0,04079	-0,00133
B_4 vs B_1	-0,0032	0,751	-0,02293	0,016536
B_5 vs B_1	-0,01971	0,056	-0,03989	0,000479
B_6 vs B_1	-0,00376	0,709	-0,02349	0,015972
C_2 vs C_1	0,009597	0,349	-0,01047	0,029663
C_3 vs C_1	0,016637	0,104	-0,00343	0,036703
C_4 vs C_1	0,012044	0,239	-0,00802	0,032111
C_5 vs C_1	0,008853	0,387	-0,01121	0,02892
C_6 vs C_1	0,004437	0,665	-0,01563	0,024503

© 2026 Mocellin D et al. *JAMA Network Open*.

eTable 11. Students group final evaluation metrics.

Outcomes	Group A (n=14)	Group B (n=14)	Group C (n=14)	Total (n=42)	p-value
PIP, mean (SD), cmH_2O	26.1 (2.6)	28.0 (2.5)	26.0 (2.8)	26.7 (2.6)	0.139
BPM, mean (SD)	42.7 (6.7)	45.1 (4.4)	46.9 (6.4)	44.9 (5.8)	0.235
Final tutor score, mean (SD) out of 12	10.6 (0.7)	10.4 (0.7)	10.3 (0.6)	10.4 (0.7)	0.294
Knowledge evaluation, mean (SE) out of 10	Pre = 8.29 ± 0.17<br>Post = 8.88 ± 0.13				p<0.001* (Wilcoxon test)

PIP = peak inspiratory pressure; BPM = breaths per minute; SD = standard deviation. SE = standard error. *Statistically significant at p < 0.05.

eTable 12. Health professionals group final evaluation

Outcomes	Group A (n=12)	Group B (n=13)	Group C (n=12)	Total (n=37)	p-value
PIP, mean (SD), cmH_2O	27.1 (4.1)	30.1 (7.9)	28.5 (3.4)	28.6 (5.1)	0.113
BPM, mean (SD)	42.8 (7.0)	39.9 (8.3)	48.8 (8.9)	43.8 (8.1)	0.038*
Final tutor score, mean (SD) out of 12	10.1 (0.5)	10.7 (1.0)	9.8 (1.3)	10.2 (0.9)	0.087
Knowledge evaluation, mean (SE) out of 10	Pre = 8.65 ± 0.17<br>Post = 9.14 ± 0.13				P=0.030* (Wilcoxon test)

PIP = peak inspiratory pressure; BPM = breaths per minute; SD = standard deviation. SE = standard error. *Statistically significant at $p < 0.05$.

© 2026 Mocellin D et al. *JAMA Network Open*.

eTable 13. Post hoc Bonferroni test for BPM

	Group A	Group B
Group B	-2.84	
	1.000	
Group C	5.99	8.83
	0.259	0.036*

© 2026 Mocellin D et al. *JAMA Network Open*.

eTable 14. Pretraining sociodemographic survey

**Age**					I don't want to answer
**Sex**	Male	Female			I don't want to answer
**Civil status**	Single	Married / engaged			I don't want to answer
**Faculty**	Midwifery	Nursing	Neonatology	Other	I don't want to answer
**Year of studies (for students)**	1	2	3	Not apply	I don't want to answer
**Year of experience (for experts)**	<5	5-10	+10	Not apply	I don't want to answer
**Prior hands-on training activities**	Yes	No			I don't want to answer
**Evaluate your household economic sustainability at the end of the month**	Well	Quite well	Not well		I don't want to answer
**Work experience prior to current studies/work**	Yes in healthcare	Yes but not in healthcare	No		I don't want to answer
**Interest in participating in this training course**	Yes	No			I don't want to answer
**Satisfaction about the frontal lessons**	1	2	3		I don't want to answer

eTable 15. Pre- and posttraining knowledge assessment

Instructions – Write **True** for correct statements and **False** for incorrect statements.

1. It is not necessary to test the functionality of the AMBU bag and mask before use if the device appears to be working properly. ___________________
2. In case of no chest rise during ventilation, it is helpful to improve the mask seal, check the newborn's head position and consider suctioning if needed. ___________________
3. Neonatal emergency ventilation should be performed at a rate of 80-100 breaths per minute, especially if the newborn's heart rate is less than 60 beats per minute. ___________________
4. During ventilation, it is crucial to monitor the newborn's muscle tone, activity, and skin color to assess the effectiveness of the procedure. ___________________
5. The correct head position for the newborn during ventilation is full neck extension. ___________________
6. The newborn's pulse should be monitored regularly during ventilation to assess the response to the intervention. ___________________
7. Ventilation is the most important step of neonatal resuscitation, more than cardiac massage. ___________________
8. Effective ventilation can be confirmed if the newborn shows an improvement in muscle tone and starts breathing spontaneously. ___________________
9. If ventilation does not improve the newborn's condition, the technique should be repeated without assessing the AMBU bag functionality or the mask size. ___________________
10. In the case of a heart rate below 60 beats per minute, chest compression should be started. ___________________

Total: ___ / 10 points

© 2026 Mocellin D et al. *JAMA Network Open*.

eTable 16. Tutor quantitative evaluation sheet**.** This sheet, which was developed by the expert external tutor who conducted the final assessment, is based on international guidelines for neonatal ventilation; however, it has not been formally validated.

Tutor evaluation sheet

Evaluator Name: ______________________
Evaluator Role: ______________________
Date: _______________
ID participant:______________

Each step is evaluated on a scale: 0 = Poor, 0.5 = Average, 1 = Good

**Item number**	**Procedure steps**	**Evaluation**
1	Gather the necessary materials for neonatal resuscitation: AMBU bag, penguin, appropriately sized mask, warm tissues, gloves, stethoscope	○ 0 Point<br>○ 0.5 Point<br>○ 1 Point
2	Put on gloves	○ 0 Point<br>○ 0.5 Point<br>○ 1 Point
3	Test the AMBU bag and mask for functionality and leaks before use	○ 0 Point<br>○ 0.5 Point<br>○ 1 Point
4	Ensure the newborn is warm and dry, position the neck slightly extended to keep the airway open, check and clear the airway if needed and provide gentle stimulation if the baby is not breathing	○ 0 Point<br>○ 0.5 Point<br>○ 1 Point
5	Position the newborn correctly with a slight head tilt	○ 0 Point<br>○ 0.5 Point<br>○ 1 Point
6	Ensure the operator is positioned at the newborn's head, directly in front to maintain optimal control and clear visibility of airway and chest	○ 0 Point<br>○ 0.5 Point<br>○ 1 Point
7	Ensure a good seal of the mask on the newborn's face using a C grip	○ 0 Point<br>○ 0.5 Point<br>○ 1 Point
8	Give 30-60 ventilation breaths per minute, ensuring just enough volume to see chest rise	○ 0 Point<br>○ 0.5 Point<br>○ 1 Point
9	Continuously evaluate the newborn during ventilation for chest movement, breathing and overall condition	○ 0 Point<br>○ 0.5 Point<br>○ 1 Point
10	Check the newborn's pulse regularly (using stethoscope or by feeling for cord pulsation)	○ 0 Point<br>○ 0.5 Point<br>○ 1 Point
11	Ensure appropriate support systems are available (e.g., oxygen, adrenaline, intubation) if the newborn requires advanced life support	○ 0 Point<br>○ 0.5 Point<br>○ 1 Point
12	Operator demonstrates knowledge of appropriate actions based on heart rate: aware of when to continue ventilation, when to seek advanced support and when to discontinue ventilation	○ 0 Point<br>○ 0.5 Point<br>○ 1 Point

Total: ___ /12 points

© 2026 Mocellin D et al. *JAMA Network Open*.

eTable 17. Posttraining TAM survey

General comments							
Which group were you part of during the training course?	Group A	Group B	Group C				
Are you a student or an expert health professional?	Student	Expert					
Overall satisfaction about the training with the simulator	Very poor	Poor	Average	Good	Excellent		
Satisfaction with your performance during the training course	Very poor	Poor	Average	Good	Excellent		
Technology Acceptance Model (TAM)							
**Perceived Usefulness (PU)**	Extremely disagree	Quite disagree	Slightly disagree	Neither	Slightly agree	Quite agree	Extremely agree
Using this simulator enables me to perform neonatal ventilation more correctly (PU1)							
Using this simulator improves my competence in the ventilation procedure (PU2)							
Using this simulator in my training enhances my confidence and self-awareness (PU3)							
Using this simulator makes it easier to do my job (PU4)							
I have found this simulator useful (PU5)							
**Perceived Easy-of-Use (PEU)**	Extremely disagree	Quite disagree	Slightly disagree	Neither	Slightly agree	Quite agree	Extremely agree
Learning how to use this simulator was easy (PEU1)							
I found it easy to handle this simulator as I wanted (PEU2)							
It was clear and understandable how to use the simulator (PEU3)							
It was easy for me to become skilful at using this simulator (PEU4)							
This simulator is easy to be used (PEU5)							
**Behavioural Intention (BI)**	Extremely disagree	Quite disagree	Slightly disagree	Neither	Slightly agree	Quite agree	Extremely agree
I was motivated in improving my professional skills by using this simulator (BI1)							
I would recommend the use of this simulator in ventilation training (BI2)							
**Fidelity of the simulator (FI)**	Extremely disagree	Quite disagree	Slightly disagree	Neither	Slightly agree	Quite agree	Extremely agree
The simulator faithfully reproduces the anatomy of the real baby (FI1)							
The simulator allows you to faithfully execute all the medical procedures necessary for performing the ventilation procedure (FI2)							
I believe the material the simulator is made of faithfully replicates human tissues and response to airflow (skin and airways resistances) (FI3)							

We would like to clarify that the terms “slightly” and “quite” were intended to provide a nuanced gradation of agreement and disagreement. Specifically, “slightly” represented a minor leaning toward one side of the neutral midpoint, whereas “quite” indicated a more firm, yet not absolute, position of agreement or disagreement.

© 2026 Mocellin D et al. *JAMA Network Open*.

eTable 18. Structural Model Results for the PLS-SEM of the TAM Survey

**Latent variable**	**PU**	**PEU**	**BI**
**Students**			
PU			0.524
P value	NA	NA	.01
PEU	0.733		
P value	.002	NA	NA
FS	0.113	0.797	0.234
P value	.62	<.001	.20
R^2	0.666	0.627	0.474
**Health professionals**			
PU			0.357
P value	NA	NA	.03
PEU	0.476		
P value	.01	NA	NA
FS	0.227	0.734	0.532
P value	.23	<.001	<.001
R^2	0.403	0.526	0.609

Abbreviations: FS, fidelity of the simulator; NA, not applicable; PEU, perceived ease-of-use; PLS-SEM, partial least squares structural equation model; PU, perceived usefulness; TAM, Technology Acceptance Model.

© 2026 Mocellin D et al. *JAMA Network Open*.
