# Supplementary material for: Neonatal Ventilation Training Using a Low-Cost, High-Fidelity Simulator in a Low-Resource Setting: A Randomized Clinical Trial
Source: JAMA Netw Open. 2026 Sep 14;9(9):e2633261. doi: 10.1001/jamanetworkopen.2026.33261 (PMC13576422; doi:10.1001/jamanetworkopen.2026.33261)
Supplement: Supplementary file 3 [file jamanetwopen-e2633261-s003.pdf]

Data Sharing Statement

Data

Additional Information: ClinicalTrials.gov, https://clinicaltrials.gov/study/NCT07665164?tab=study, ClinicalTrials.gov ID NCT07665164
Data available: Yes
Data types: Deidentified participant data
How to access data: Data are available on request to davide.mocellin@santannapisa.it
When available: With publication

Supporting Documents

Document types: None

Additional Information

Who can access the data: Researchers whose proposed use of the data has been approved.
Types of analyses: For any purpose.
Mechanisms of data availability: With a signed data access agreement after approval of a proposal
Any additional restrictions: None
